# Predictive Sequence Analysis of the *Candidatus* Liberibacter asiaticus Proteome

**DOI:** 10.1371/journal.pone.0041071

**Published:** 2012-07-18

**Authors:** Qian Cong, Lisa N. Kinch, Bong-Hyun Kim, Nick V. Grishin

**Affiliations:** 1 Howard Hughes Medical Institute, University of Texas Southwestern Medical Center, Dallas, Texas, United States of America; 2 Department of Biophysics, University of Texas Southwestern Medical Center, Dallas, Texas, United States of America; University of Alberta, Canada

## Abstract

*Candidatus* Liberibacter asiaticus (*Ca.* L. asiaticus) is a parasitic Gram-negative bacterium that is closely associated with Huanglongbing (HLB), a worldwide citrus disease. Given the difficulty in culturing the bacterium and thus in its experimental characterization, computational analyses of the whole *Ca.* L. asiaticus proteome can provide much needed insights into the mechanisms of the disease and guide the development of treatment strategies. In this study, we applied state-of-the-art sequence analysis tools to every *Ca.* L. asiaticus protein. Our results are available as a public website at http://prodata.swmed.edu/liberibacter_asiaticus/. In particular, we manually curated the results to predict the subcellular localization, spatial structure and function of all *Ca.* L. asiaticus proteins (http://prodata.swmed.edu/liberibacter_asiaticus/curated/ ). This extensive information should facilitate the study of *Ca.* L. asiaticus proteome function and its relationship to disease. Pilot studies based on the information from our website have revealed several potential virulence factors, discussed herein.

## Introduction

*Candidatus* Liberibacter asiaticus (*Ca.* L. asiaticus) is a Gram-negative *Alphaproteobacterium*. It is closely associated with Huanglongbing (HLB, also called citrus greening), one of the most severe worldwide diseases of citrus. The ranking *Candidatus* is assigned to this bacterium because it cannot be maintained in bacterial culture. In nature, the bacterium is transmitted among citrus plants by the piercing-sucking insect, Asian citrus psyllid (*Diaphorina citri Kuwayama*) and it resides in the phloem tissue of citrus plants. Infected plants gradually develop symptoms such as yellow leaves, premature defoliation and aborted fruits, followed by the eventual death of the entire plants [1], [2]. It is hypothesized that *Ca.* L. asiaticus infection could induce over-accumulation of callose in plant plasmodesmata pore units and sieve pores, inhibiting phloem transport and contributing to HLB symptoms [3], [4], [5].

Ever since HLB was described, efforts have been devoted to understanding the plant response to the infection [6], [7], and to diagnosing [8], [9] and controlling the disease [10], [11], [12]. However, a fundamental understanding of the HLB mechanism or an ultimate way to treat the disease has yet to manifest. This lack of accomplishment is due in part to the limited success in culturing the bacterium [13], which makes carrying out experiments directly on *Ca.* L. asiaticus a challenge.

In 2009, the complete genome sequence of *Ca.* L. asiaticus was obtained [14] and verified [15], allowing researchers to study *Ca.* L. asiaticus proteins *in vitro* or through heterologous expression. Through such experiments, the function of a hypothetical ADP/ATP translocase has been verified [16] and a moderate inhibitor of the predicted *secA* gene product has been identified [17]. These findings demonstrate the possibility of understanding and controlling HLB at the molecular level. Given the genome sequence, computational analysis combined with manual curation can stimulate such research by predicting the structure and function of *Ca.* L. asiaticus proteins, identifying potential virulence factors and selecting drug targets to specifically inhibit the bacterium.

The *Ca.* L. asiaticus genome is highly reduced relative to other bacteria in the order *Rhizobiales*, likely related to its intracellular lifestyle [18]. Gene prediction and annotation pipeline from National Center for Biotechnology Information (NCBI) [19] and the Rapid Annotations using Subsystems Technology (RAST) server [20], [21] have predicted 1,233 protein-coding genes in the entire genome. This relatively small genome size allows careful analysis of all the *Ca.* L. asiaticus proteins *in silico*. Protein sequence analysis relies heavily on detection of homologs [22], [23]. The 3D structures of homologous proteins provide templates for structure modeling, and the function annotations of close homologs (orthologs) can be transferred in most cases to the protein of interest. Meanwhile, in the absence of confident homologs, other evidence can provide hints to protein function, including the presence of certain functional motifs, the predicted 3D structure, the genomic context, the phylogenetic distribution, the known physical interaction, functional association between proteins and detection of local sequence features such as signal peptides (SPs) and transmembrane helices (TMHs) [24], [25].

Here we report computational analysis followed by partial manual curation of the *Ca.* L. asiaticus proteome. Information from various databases was gathered for each protein and essential sequence features, such as SPs and TMHs, were predicted. Moreover, the evolutionarily related proteins, protein families, protein structures and domains were detected for each *Ca.* L. asiaticus gene product by multiple procedures. Results from these bioinformatics analyses were compiled as a website at http://prodata.swmed.edu/liberibacter_asiaticus/. On the basis of this information, in-depth manual analysis can be performed to predict subcellular localization, validate function predictions, generate structural models, analyze domain architectures and, most importantly, identify potential effectors of this pathogen and targets for treating HLB. To illustrate the potential applications of the database, we predicted the 3D structure and function of each *Ca.* L. asiaticus protein (summarized in an additional website at http://prodata.swmed.edu/liberibacter_asiaticus/curated/). Specifically, we revealed several potential virulence factors that may be helpful to understand and control HLB from analyzing duplicated proteins and the proteins whose closest homologs are from phylogenetically distant species.

## Methods

### Construction of the Website

All the sequences of *Ca.* L. asiaticus proteins predicted by NCBI gene prediction pipeline were downloaded from the GenBank database (ftp://ftp.ncbi.nih.gov/genbank/genomes/Bacteria/Candidatus_Liberibacter_asiaticus_psy62_uid29835) and additional hypothetical proteins that were detected by the SEED (Genome annotation web service on the basis subsystems, http://pseed.theseed.org/seedviewer.cgi) but missed by NCBI were added. The relevant information about each protein was obtained from NCBI (http://www.ncbi.nlm.nih.gov/nuccore/CP001677), the SEED, and Kyoto Encyclopedia of Genes and Genomes [26] (KEGG, http://www.genome.jp/kegg-bin/show_genomemap_top?org_id=las). For each protein, computational analysis was performed as follows.

First, we predicted the local sequence features (listed in Table 1) of each protein by multiple predictors with default parameters. Second, we detected their close homologs by 2 iterations of PSI-BLAST [27] from the non-redundant database (NR, 05/22/2011) with e-value 0.005 as cutoff. Out of the PSI-BLAST 2^nd^ iteration hits, two sets of representative sequences were selected. The first representative set is filtered by more than 40% alignment coverage and less than 90% sequence identity, while the second set is selected with more than 40% alignment coverage and less than 70% sequence identity as cutoff. These representatives were used to construct sequence profiles based on BLAST alignments and to calculate the positional conservation indices by AL2CO [28]. Third, related protein families were detected from Conserved Domain Database (CDD) [29], [30], [31], [32], [33], [34] by RPS-BLAST (e-value cutoff 0.005) [35] and HHsearch (probability cutoff 90%) [36]. Fourth, to detect evolutionarily related protein structures and reveal domain architectures, we used three protocols: 1) PSI-BLAST (e-value cutoff 0.005) against the NR database (05/22/2011), starting from the sequence profiles built by the buildali.pl script in the HHsearch package, 2) RPS-BLAST (e-value cutoff 0.005) and 3) HHsearch (probability cutoff 90%) against the 70% sequence identity representatives of all PDB entries (up to Jun, 2011), the Structure Classification of Proteins (SCOP, version 1.75) database [37] and the Molecular Modeling DataBase (MMDB, up to Jan, 2011) from NCBI [38], with each single protein sequence as a query. All the results and useful information from other resources (NCBI, SEED and KEGG) were integrated and represented in a web page. All the web pages were assembled to establish a public website for the *Ca.* L. asiaticus proteome.

**Table 1:** The Predicted Local Sequence Features.

Feature	Programs Used For The Prediction	Implication
**Secondary structure**	PSIPRED (v2.0) [81] and SSPRO (v4.0) [82]	assist 3D structure and domain boundary prediction
**Disordered or flexible region**	DISEMBL (v1.5) [83], DISPRO (v1.0) [84] and DISOPRED (v2.0) [85]	assist 3D structure modeling and indicate the domain boundaries
**Transmembrane helix**	TMHMM (v2.0), TOPPRED (v2.0), HMMTOP (v2.0), MEMSAT (v3.0), MEMSATSVM and Phobius	predict subcellular localization; provide hints to the protein function. predict the topology of membrane proteins
**Signal peptide**	SignalP (v3.0), Phobius and MEMSATSVM	predict secreted proteins that could potentially be virulence factors
**Low-complexity**	SEG [86]	Reveal false positive hits of homology search caused by matching of low-complexity region
**Coiled coil**	COILS [87]	reveal false positive hits of homology search caused by matching of non-homologous coiled coils
**Conservation**	PSI-BLAST, AL2CO	reveal essential residues for the folding and function of a protein

### Application of the Website

Based on the information on our website, we manually assigned function to each protein and selected templates to build a structural model by homology modeling using MODELLER [39]. Functional annotations were mainly based on close relationships to known proteins and protein families. These relationships were verified on the one hand by the statistical significance, coverage and alignment quality, and on the other hand by the consensus between different methods and annotations made by other databases. In cases where agreement between methods was lacking or statistical support was marginal, identification of conserved sequence motifs, inspection of predicted structure and clustering of homologous proteins by CLANS [40] were applied to assist function predictions.

Homologous proteins within the *Ca.* L. asiaticus proteome were identified among BLAST hits (e-value cutoff 0.005). Homologous groups within the *Ca.* L. asiaticus proteome were established manually in a single-linkage manner [41] on the basis of BLAST results, requiring grouped proteins to have similar predicted functions. All the homologous groups with more than one protein were studied manually. From these groups, potential virulence factors were identified and analyzed in detail. In addition, the taxonomy information of the best BLAST hits (e-value cutoff 0.005) of each *Ca.* L. asiaticus protein was examined. Proteins with their best hits from organisms other than *Alpharoteobacteria* were then investigated carefully to identify potential horizontal gene transfer (HGT) events and virulence factors.

## Results and Discussion

### Description of the Website

The results of computational analysis of all 1,233 *Ca.* L. asiaticus proteins are presented as a website at http://prodata.swmed.edu/liberibacter_asiaticus/. The proteins are sorted by the genomic loci of their coding genes to allow easy navigation of their genomic context. A web page is devoted to each protein, containing the following information.

#### Section I. Basic information (illustrated in Fig. 1A)

This section provides relevant information from and links to other databases. Several existing annotations were listed, including: gene description from NCBI (definition line in NCBI Protein Database), COG prediction (from NCBI, based on homologous relationship to protein families in the Cluster of Orthologous Groups (COG) database), KEGG prediction (annotation in the KEGG database) and the SEED prediction (annotation in the SEED database).

**Figure 1:**
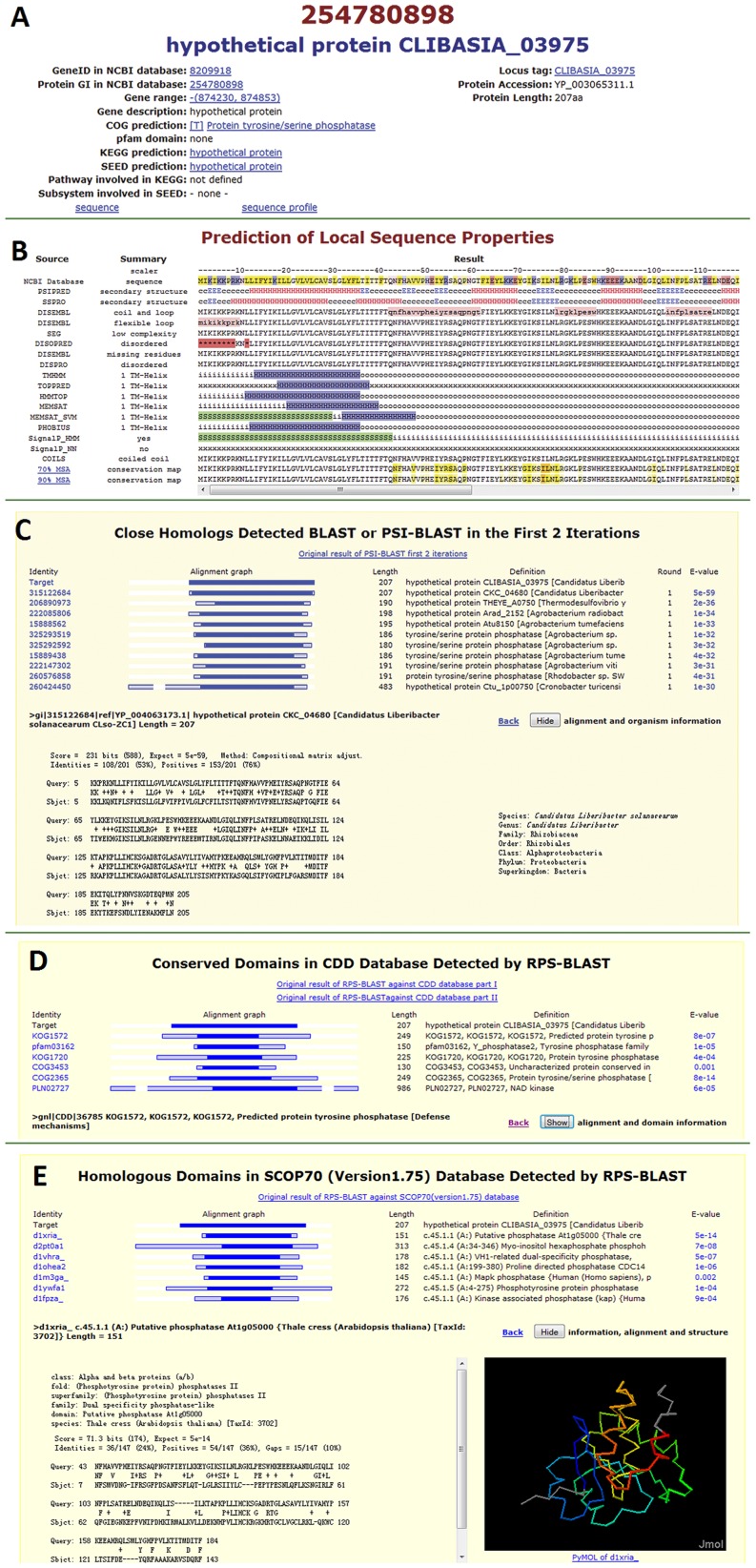
Illustration of the webpage. (A) Section I: basic information with function predictions from different resources and links to other databases. (B) Section II: local sequence feature prediction. It contains the following information: (1) sequence (highlighted according to the property of amino acid) from NCBI database; (2),(3) secondary structure prediction by PSIPRED and SSPRO (H: α helix, E: β strand, C: coils); (4) Coil and loop (highlighted in pink) prediction by DISEMBL; (5) Flexible loop (highlighted in pink) prediction by DISEMBL; (6) Low complexity region (highlighted in light red) prediction by SEG; (7)-(9): Disordered region (highlighted in red) prediction by DISPRED, DISEMBL and DISPRO; (10)-(15) Transmembrane helix (highlighted in blue) prediction by TMHMM, TOPPRED2, HMMTOP, MEMSAT, MEMSATSVM, Phobius; (14)-(17) Signal Peptide (highlighted in green) prediction by MEMSATSVM, Phobius, SignalP Hidden Markov Model mode and SignalP Neural Network mode; (18) Coiled coils (highlighted in yellow) prediction by COILS; (19),(20) Sequence colored by conservation (highlighted from white, through yellow to dark red as the level of conservation increases) computed on the Multiple Sequence Alignment of homologous proteins filtered by 70% or 90% sequence identity. (C) Section III: top 10 homologs detected by BLAST or 2 iterations of PSI-BLAST are listed. For each hit, the alignment and the species associated with the hit are provided. (D) Section IV: homologous protein families and conserved domains detected by RPS-BLAST. The confident hits detected by certain method are listed and the relative information of each protein family and its alignment to the *Ca.* L. asiaticus protein can be retrieved. (E) Section VI: evolutionary related protein domains detected by RPS-BLAST in SCOP database. It includes a table summarizing all confident hits, followed by details of each hit, including its hierarchy in SCOP, the alignment and the 3D structure visualized in Jmol.

#### Section II. Prediction of local sequence features (illustrated in Fig. 1B)

Local sequence properties, such as predicted secondary structures and disordered regions, are helpful for predicting 3D structures, whereas, SP and TMH predictions are suggestive of protein localization and function. This section summarizes prediction of local sequence features (listed in Table 1). The result from each predictor is represented as a string consisting of each residue’s predicted status and this string is aligned to the original protein sequence for convenient comparison.

#### Section III. Close homologs (illustrated in Fig. 1C)

Close homologs usually share similar functions inherited from a common ancestor, which is the basis for function prediction. In addition, the phylogenetic distribution of closely related proteins provides hints about the evolutionary history and reveals HGT events. HGT has a profound impact on the evolution of bacterial pathogens and it is a common mechanism to gain virulence-associated genes [42]. Thus, the 10 closest homologs detected by BLAST or 2 iterations of PSI-BLAST (e-value cutoff 0.005) are provided in ranked order. On top of this section, a summary line for each hit provides links to relevant information, including the NCBI gi linked to the corresponding page at NCBI and a bar graph alignment overview linked to the pairwise BLAST or PSI-BLAST alignment and the taxonomy information, which is on the bottom of this section. Moreover, we specifically detected and reported homologs (if any) from *Ca.* L. asiaticus so that these duplicated genes can be compared and analyzed together.

#### Section IV: Homologous protein families (illustrated in Fig. 1D)

Protein classification and the extensive information gathered for protein families in databases are valuable resource for functional annotation. In this section, we listed related protein families and conserved domains identified by RPS-BLAST (e-value cutoff 0.005) and HHsearch (probability cutoff 90%) in ranked order. Information is presented in similar format to that described in section III, with a summary of hits at the top and detailed alignments and descriptions of protein families listed at the bottom.

#### Section V. Homologous structures and domains (illustrated in Fig. 1E)

Homology modeling remains the most reliable and effective way to predict protein 3D structure [43], [44]. This section is designed for structure modeling. Homologous structures and structure domains detected by PSI-BLAST (e-value cutoff 0.005), RPS-BLAST (e-value cutoff 0.005) and HHsearch (probability cutoff 90.0%) are presented in similar format as described in Section III. For each hit, the alignment and the corresponding structure displayed by Jmol (an open-source Java viewer for chemical structures in 3D, http://www.jmol.org/) can be easily retrieved. These protein structures can be used as templates to generate a 3D structural model. For structure domains detected in SCOP, we provide their classification hierarchy, which places them in an evolutionary context and suggests similarities to other proteins.

### Proteome-wide Prediction for *Ca.* L. asiaticus

With the information from our website, we performed manual analysis to predict the spatial structure and function of each protein, and the results are available at http://prodata.swmed.edu/liberibacter_asiaticus/curated/. In the genome sequence of *Ca.* L. asiaticus, the gene prediction pipeline from NCBI and SEED detected 1,233 protein coding genes, with 1,046 in common. In addition, 59 proteins whose corresponding genes are identified by a single pipeline display confident homology to other proteins in the NR database. We consider these 1,105 protein coding genes to be confidently predicted. However, the products of the remaining 128 genes exhibit a relatively small size (usually less than 60 residues), include low-complexity sequence, lack similarity to any known proteins, and are inconsistently predicted by the gene prediction pipelines. These genes may represent falsely predicted open reading frames and they are not considered in the following analysis.

#### Prediction and manual assessment of subcellular localization

We combined the results of computer programs and manual curation to identify potential transmembrane and extracytoplasmic proteins. We applied 6 TMH predictors (TMHMM [45], HMMTOP [46], TOPPRED [47], MEMSAT [48], MEMSAT_SVM [49] and Phobius [50]) and two of them (MEMSAT_SVM and Phobius) detect SPs that are likely to be processed by the Sec complex. In addition, we used the well established SignalP3.0 [51], which contains both Hidden Markov Model (SignalP_HMM) and Artificial Neural Network (SignalP_NN) modes for SP prediction. These automatic methods are generally based on the local properties of protein sequences or sequence profiles, resulting in a considerable rate of false predictions. Consequently, we manually inspected all the proteins that are predicted to have TMHs or SPs by any automatic predictors we applied. This broad inclusion can help lower the false negative rate. At the same time, to control the false positive rate, we integrated several lines of evidence, including consensus between predictors, predicted 3D structure (to rule out buried hydrophobic segments in known cytoplasmic proteins) and function (to identify proteins and protein domains known to function outside the cytoplasm or in the membrane), features of a protein’s orthologs (to validate if the SPs and TMHs can be constantly predicted in a orthologous group) and specific information about secretion machineries of *Ca.* L. asiaticus.

Periplasmic and extracellular proteins are generally targeted to their specific subcellular compartments via protein secretion systems. Gram-negative bacteria possess 6 classic protein secretion systems. Type II and Type V Secretion Systems transport proteins from periplasm to extracellular space. Their function requires Sec or Tat machinery to translocate proteins from cytoplasm to periplasm. In contrast, Type I, Type III, Type IV and Type VI Secretion Systems can directly export proteins from cytoplasm to extracellular space and thus do not depend on Sec or Tat [52]. *Ca.* L. asiaticus likely harbors all the basic components of the Sec machinery and the Type I Secretion System (TISS), an ABC-type protein transporter [53].

A substrate of the Sec complex can be recognized by an N-terminal SP, which is a hydrophobic α-helical segment flanked by a positively charged short region at its N-terminus and several polar residues at its C-terminus that could be cleaved by the Sec machinery. We manually examined all 218 proteins that were predicted to have SPs by any automatic method to identify extracytoplasmic proteins. After integrating additional evidence, we hypothesize that 86 proteins (marked in Supplementary Table S1 at http://prodata.swmed.edu/congqian/paper/supplement_table_S1.pdf) with predicted SPs are likely secreted from cytoplasm to periplasm via the Sec machinery. Predictions and supporting evidence for each protein are listed in Supplementary Table S2 at http://prodata.swmed.edu/congqian/paper/supplement_table_S2.pdf.

Many proteins from the initial list of 218 candidates were excluded due to the following reasons: (1) the SP cannot be consistently predicted (predicted by only 1 out of 4 methods); (2) the protein is predicted to have multiple TMHs, such as the sensory box/GGDEF family protein (locus: CLIBASIA_01765; gi: 254780468); (3) the confidently predicted function of the protein suggests its localization in the inner membrane or cytoplasm, for example, the ribosomal protein L35, which is predicted to have a SP by 3 out of 4 predictors applied; (4) close homologs likely lack SPs. It is important to note that transmembrane proteins might have SPs at their N-termini, although such cases are not common in bacteria [54]. Nevertheless, these proteins will likely be localized in the inner membrane by other TMHs regardless whether the SPs will be cleaved or not.

However, this bacterium and the other congener (*Candidatus* Liberibacter solanacearum) appear to lack classic components of the Sec-dependent Type II and Type V Secretion Systems. This does not mean that these 86 proteins must all locate in periplasm or outer membrane. Instead, *Ca.* L. asiaticus might adopt some noncanonical mechanism to route proteins across the outer membrane. One possible mechanism would be to “hijack” the *flp* pilus assembly system present in this bacterium, which is evolutionarily related and functionally similar to the Type II Secretion System [55]. It is also possible that some β-barrel proteins in the outer membrane have adopted the ability to transport proteins to the extracellular space [56]. Finally, some of these proteins might be secreted in outer membrane vesicles as discovered in other Gram-negative bacteria [57], [58]. In summary, these proteins putatively secreted by Sec could localize in the periplasm, outer membrane or extracellular space, and they contain candidates for virulence factors of this pathogen.

Proteins without SPs can be secreted in Sec-independent manners. We detected these proteins by their homology to known substrates of Sec-independent secretion systems and their genomic loci. *Ca.* L. asiaticus possesses the TISS. Substrates of TISS are characterized by calcium binding glycine-rich repeats (GGXGXDXXX) in their sequences, which can adopt peculiar β-sandwich or β-roll structures [59]. Such signature motifs in TISS substrates are grouped in cluster 2931 (COG2931) in the COG database. Among all *Ca.* L. asiaticus proteins, only a predicted serralysin (locus: CLIBASIA_01345, gi: 254780384) shows confident similarity to COG2931. This relationship is further supported by the sequence pattern and predicted 3D structure of this putative serralysin. Moreover, the coding gene of this protein locates next to the TISS locus in the genome. We hypothesize that it can be secreted to extracellular space directly through TISS, where it may act as a virulence factor in the host. Specifically, this protein is a protease homolog; it may interfere with proteins participating in plant immune responses.

In addition, the flagellar assembly and *flp* pilus assembly machineries are also likely preserved in this bacterium and many flagellar and *flp* pilus components without SPs can be secreted through them [60]. In *Ca.* L. asiaticus, 10 flagellar components and 10 *flp* pilus components are likely secreted to the extracytoplasmic space through their dedicated assembly machineries (listed in Supplementary Table S2) as suggested by studies on their orthologs in other organisms [61], [62].

576 *Ca.* L. asiaticus proteins are predicted to have potential TMHs by any of the automatic methods were studied manually. We considered consensus between TMH predictors, topology of the identified 3D structure templates, predicted function and presence of manually validated SPs (judgment and evidence listed in Supplementary Table S3 at http://prodata.swmed.edu/congqian/paper/supplement_table_S3.pdf). As a result, we suggest that184 *Ca.* L. asiaticus proteins are targeted in the inner membrane (marked in Supplementary Table S1). The initial list of potential transmembrane proteins contains a large portion of false positives due mainly to the presence of a hydrophobic segment buried in the structural core of a cytoplasmic protein. HMMTOP and TOPPRED generated relatively high false positive rates in this proteome-wide study, likely due to their emphasis on predicting the topology of a given transmembrane protein rather than distinguishing membrane proteins from cytoplasmic ones. In addition, TMH predictors that do not distinguish TMHs from SPs (TMHMM, HMMTOP, TOPPRED and MEMSAT) frequently recognize a SP as a TM due to their similar sequence properties.

**Figure 2:**
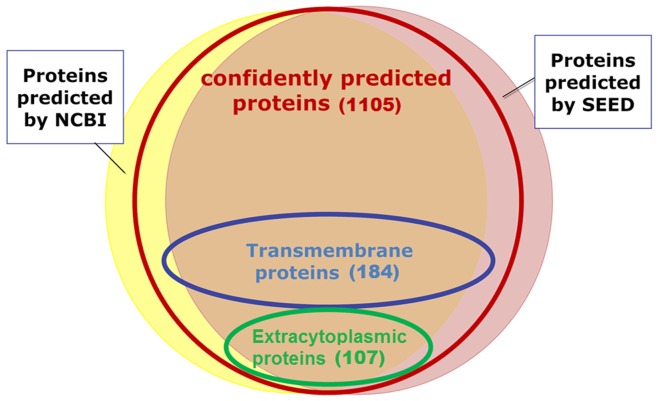
Venn diagram of the predicted protein coding genes by different methods in the *Ca.* L. asiaticus genome. The yellow disk represents the set of protein coding genes identified by NCBI and the pink disk stands for the set of protein coding genes predicted by the SEED. The red, blue and green circle includes all confidently predicted protein coding genes, transmembrane proteins and secreted proteins via Sec in the proteome after manual inspection.

In summary, we hypothesize that 86 proteins are secreted via the Sec machinery and 21 without SPs are likely targeted to the extracytoplasmic space through Sec-independent mechanisms. In addition, 184 proteins likely locate in the inner membrane of this Gram-negative bacterium (shown in Fig. 2). Comprehensive information from our website allows us to correct mistakes of computer programs and to generate more reliable hypotheses about a protein. However, due to the limited information available for some proteins and the limitation that we only curated proteins with predicted SPs or TMHs, it is possible that erroneous predictions still exist even after careful manual study.

#### Prediction of 3D structure and function

Confidently identified homology to known proteins or protein families allows us to predict the functions of 80.4% of all 1,105 proteins, while NCBI and SEED annotated 67.6% and 71.0% of them, respectively, or 74.1% combined. Moreover, out of the 217 proteins lacking explicit function predictions, based on our manual curation discussed above, 40 are predicted to be secreted through Sec machinery and thus function in extra cytoplasm. 49 unannotated proteins are likely to be transmembrane proteins in the inner membrane. These proteins comprise 41.0% of the unannotated proteins. Their predicted subcellular localization suggests their general function in communicating with the environment. (All function and localization predictions are listed in Supplementary Table S1).

**Figure 3:**
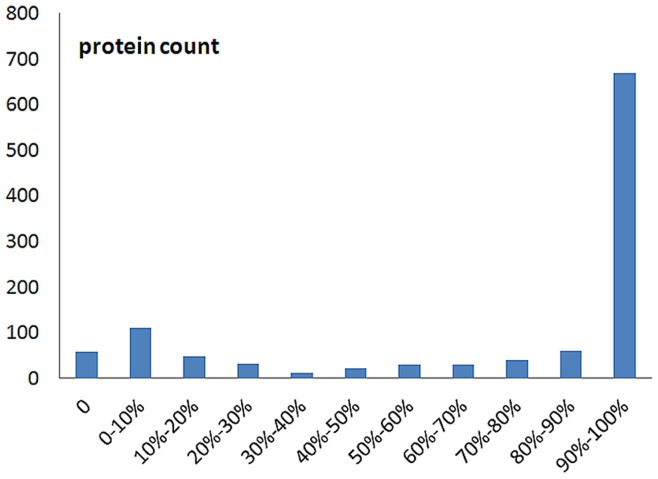
The distribution of 3D structure prediction coverage for each protein.

Another application of our website is to present putative homologous structures for template-based structure modeling. Confident templates identified by programs (HHsearch probability cutoff 90%, PSI-BLAST or RPS-BLAST e-value cutoff 0.005) and confirmed by manual curation cover 74.3% of all residues in the *Ca.* L. asiaticus proteome. In addition, some regions that appear at the boundaries of protein domains are predicted to be disordered by at least 2 predictors out of 3. These regions count for another 5.8% of all residues. At the level of individual proteins, 65.9% of all *Ca.* L. asiaticus proteins show at least 80% coverage by structure templates and disordered regions (Fig. 3). It is important to emphasize that we adopted conservative criteria for selecting structure templates, which would underestimate the number of *Ca.* L. asiaticus proteins whose 3D structures can ultimately be predicted by homology modeling.

### In-depth Studies Based on Our Website Reveal Potential Virulence Factors of *Ca.* L. asiaticus

More specific analysis of the *Ca.* L. asiaticus proteome can be performed on the basis of our website. To illustrate, we analyzed clusters of homologous proteins within the proteome and proteins with abnormal evolutionary history, placing emphasis on the identification of potential virulence factors. We define virulence factors as gene products that enable a pathogen to colonize in the host, battle with the defense system and cause damage to the host [63]. Plants exhibit pathogen-inducible defense mechanisms and the basal defense of a plant host can be elicited by pathogen-associated molecular patterns (PAMPs). Known PAMPs include bacterial lipopolysaccharide, peptidoglycan and flagellin [64]. The *Ca.* L. asiaticus proteome seems to include almost all components of the flagellar assembly, including flagellin, FliC (locus: CLIBASIA_02090, gi: 254780531), which might be able to initiate PAMP-triggered immunity (PTI) responses in citrus. Common PTI responses include callose deposition, ethylene production and induction of pathogenesis-related proteins that can stop the bacterium from further colonization [65]. The detection of accumulated callose in plasmodesmata pore units and sieve pores after *Ca.* L. asiaticus infection supports the existence of PTI in citrus. Similarly to other plant pathogens, *Ca.* L. asiaticus likely utilizes virulence factors to interfere with PTI and escape from the plant immune response. These pathogenic factors are essential for understanding the mechanism of HLB.

#### Homologous protein groups within the genome

**Figure 4:**
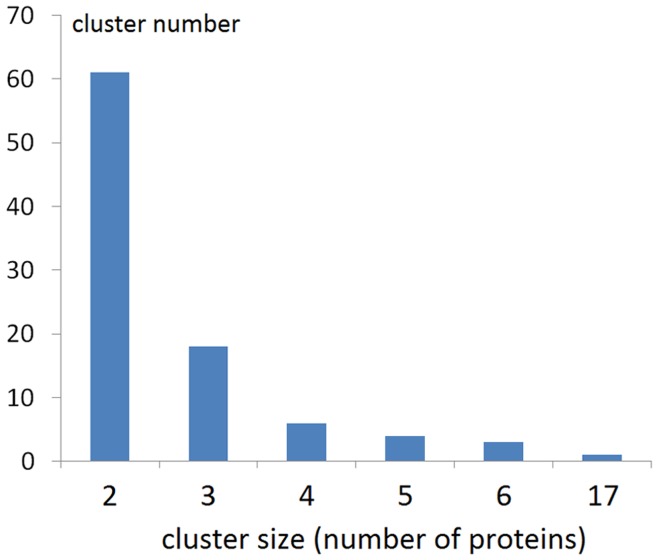
Distribution of homologous protein cluster sizes (clusters of single proteins excluded) within the *Ca.* L. asiaticus proteome.

22% of *Ca.* L. asiaticus proteins have detectable homologs by BLAST within the same proteome, which is lower than the average (31%) for bacterial proteomes of similar size (shown in Supplementary Table S4 at http://prodata.swmed.edu/congqian/paper/supplement_table_S4.pdf). Based on detectable sequence similarity by BLAST, we identified all the homologous protein clusters within the *Ca.* L. asiaticus proteome. The distribution of cluster sizes is shown in Fig. 4 (trivial clusters consisting of just one protein excluded). We further studied clusters with more than one protein and classified them into 3 categories according to our interpretations of the duplication events. (Details for each cluster are shown in Supplementary Table S5 at http://prodata.swmed.edu/congqian/paper/supplement_table_S5.pdf).

The first category is **Ancient Duplication Events during the Functional Divergence of Proteins** (colored green in Supplementary Table S5). They represent either paralogs with similar function but different specificity and partners, such as ABC-transporters, GTP-binding proteins, amino acid-tRNA synthetases, or evolutionarily related proteins that cooperate with each other in the same pathway or complex, e.g., *flp* pilus assembly proteins and NADH dehydrogenase subunits. Such phenomena as paralogous proteins cooperate with each other in a same process or participate in similar steps of different pathways are common during the function divergence of proteins [66]. The largest homologous cluster contains the ABC-transporter-type P-loop containing ATPases. The ABC-type ATPase is the largest protein family in bacteria [67] and its members mainly work together with transmembrane permeases to function as ATP-binding cassette transporters (ABC transporters) [68]. In this parasitic bacterium, they function to gain nutrition, resist harmful compounds in the environment and construct outer membrane; thus, they likely play important roles for the survival of this bacterium.

**Figure 5:**
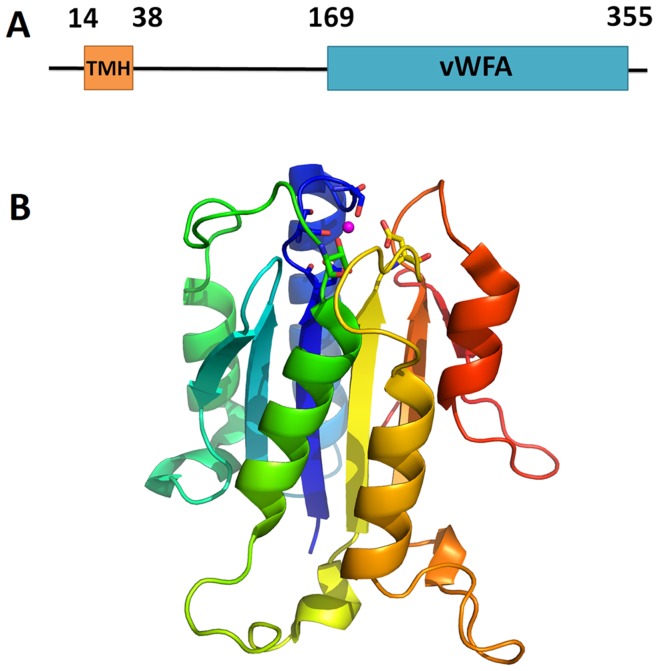
Potential virulence factor, von Willebrand factor type A domain containing protein. **(locus: CLIBASIA_03630, gi: 254780833).** (A) Domain diagram of the protein (B) Predicted structure of the protein colored in rainbow. The side-chains of the conserved residues for metal binding are shown.

**Table 2:** Duplicated proteins that are unique to *Ca.* L. asiaticus proteome.

Group	Locus	gi	Comments
I	CLIBASIA_02215	254780556	with SP, potential virulence factor
	CLIBASIA_04405	254780980	with SP, potential virulence factor
II	CLIBASIA_03915	254780886	with SP, potential virulence factor
	CLIBASIA_04530	254781005	with SP, potential virulence factor
III	CLIBASIA_04425	254780984	with SP, potential virulence factor
	CLIBASIA_05140	254781126	do not have the SP part
	CLIBASIA_04410	254780981	with SP, potential virulence factor
IV	CLIBASIA_00440+ CLIBASIA_00445	254780203+254780204	Two neighboring proteins both aligned to part of CLIBASIA_05480. It is possible they are psuedogenes
	CLIBASIA_05130+ CLIBASIA_05135	254781124+254781125	Two neighboring proteins both aligned to part of CLIBASIA_05480. It is possible they are psuedogenes
	CLIBASIA_05480	254781189	Transmembrane protein

The second category of duplicated genes is **Recent Duplication likely caused by the Integration of Bacteriophage** (colored yellow in Supplementary Table S5). This category includes protein pairs with very high sequence identity (more than 90% or even 100%), indicating recent duplication events. Last year, the sequences of the SC1and SC2 Liberibacter phages [69] that coincide with *Ca.* L. asiaticus were reported. Our analysis reveals that the current genome sequence of *Ca.* L. asiaticus str. psy62 (GenBank ID: CP001677.5) very likely harbors an integrated SC1 Liberibacter phage as supported by the following evidence. First, the SC1 Liberibacter phage genome sequence can be aligned to the *Ca.* L. asiaticus genome with over 98% identity in nucleotide sequence. Second, 42 of the *Ca.* L. asiaticus protein coding genes consecutively locate in the *Ca. L. asiaticus* genome (highlighted in green in Supplementary Table S6 at http://prodata.swmed.edu/congqian/paper/supplement_table_S6.pdf) correspond exactly to all proteins in the SC1 Liberibacter phage. Finally, one SC1 Liberibacter phage protein can be aligned to two duplicated *Ca.* L. asiaticus proteins at their N- and C-terminal halves, respectively; we hypothesize that these two proteins contain the site at which the phage integrated into the bacterial genome. Many proteins in the prophage region, such as SNF2 family DNA/RNA helicase, NAD-dependant DNA ligase, and guantylate kinase, have close homologs in the bacterial proteome. And these are likely homologous recombination events mediated by the SC1 Liberibacter phage. The proteins belonging to the integrated SC1 Liberibacter phage, especially proteins that are not related to the life cycle of the phage may contribute to the pathogenicity of *Ca.* L. asiaticus, as bacteriophages are common vectors for transmitting pathogenicity islands among bacteria [70].

**Figure 6:**
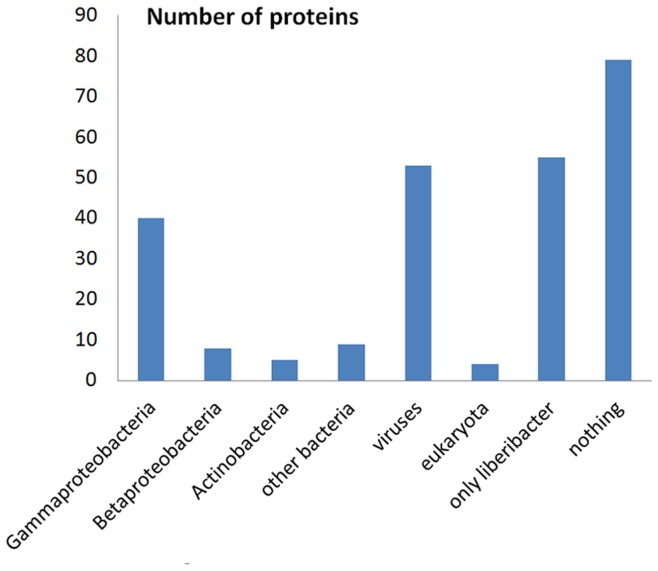
The distribution of *Ca.* L. asiaticus proteins’ closest homologs among organisms. (proteins from Liberibacter genus excluded).

The third category contains **Potential Virulence Factors** (colored red in Supplementary Table S5). These are of primary interest, and thus we classified them in a special category. Their proposed roles in bacterial pathogenicity are supported by one or several of the following evidence: (1) homology to known virulence factors; (2) presence of a SP; (3) lack of detectable homologs in other organisms, consistent with fast evolution. Multiple copies of similar virulence proteins may cooperate with each other, intensifying the pathogenicity.

One of the most unusual homologous groups consist of von Willebrand factor type A (shown in Fig. 5) (vWA) domain containing proteins. There are five such proteins in *Ca.* L. asiaticus. Out of them, only CLIBASIA_05050 (gi: 254781108) and CLIBASIA_05060 (gi: 254781110) are annotated as vWA. However, all evidence suggests that hypothetical proteins CLIBASIA_01365 (gi: 254780388), CLIBASIA_03630 (gi: 254780833) and CLIBASIA_04165 (gi: 254780934) also contain vWA domains. Starting from any protein in this group, all homology inference methods we used detect vWA domains with confident statistics (e-value below 1e-5 for RPS-BLAST and HHsearch probability above 99.8%). Every protein in this group preserves a metal ion-dependent adhesion site (MIDAS, shown in Fig. 5B), which is the signature of vWA domains. In addition, a TadE/F-like domain (pfam07811) is detected at the N-termini of these vWA domain containing proteins. TadE and TadF proteins are closely related, and they are components of the *flp* pilus assembly system. Each of them is proposed to harbor a TMH at its N-terminus that anchors it to the inner membrane of Gram-negative bacteria. The transmembrane aspartate protease, CpaA is proposed to specifically cleave this TMH and release TadE/TadF into the periplasmic space to participate in *flp* pilus assembly [71]. *Ca.* L. asiaticus is predicted to have all basic components of the *flp* pilus assembly machinery described in *Actinobacillus actinomycetemcomitans* except single-domain TadE and TadF. Thus we hypothesize that these vWA-domain-fused TadE/TadF might function in a similar way to TadE and TadF, being processed by the CpaA (locus: CLIBASIA_03080, gi: 254780729) and released to the periplasm. They might subsequently act as components for the *flp* pilus assembly. Alternatively, they might take advantage of the *flp* pilus assembly machinery (which assembles Type II secretion system), outer membrane β-barrels or membrane vesicles to enter the extracellular space. Mainly found as extracellular eukaryotic domains, vWA domains are involved in cell adhesion, migration, homing, pattern formation, and signal transduction [72], [73]. Although the function of vWA domains in bacteria is still unclear, they have been detected in some “repeat in toxin” proteins (rtx, typical virulence factors secreted by TISS) and are proposed to be virulence factors of the human pathogen, *Legionella pneumophila* [74]. Similarly, these vWA domain-containing proteins might be able to function in the extracellular space and use their MIDAS motif to interact with host proteins, thus contributing to the pathogenicity of *Ca.* L. asiaticus.

**Figure 7:**
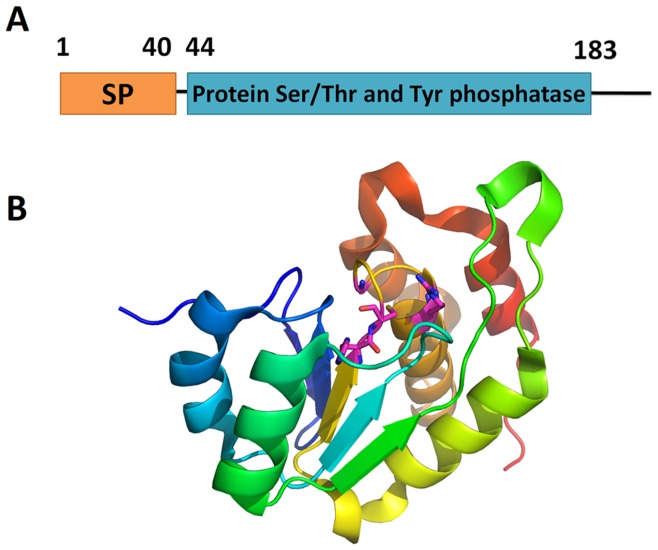
Potential virulence factor, protein serine/tyrosine phosphatase. (locus: CLIBASIA_03975, gi: 254780898). (A) Domain diagram of the protein (B) Predicted structure of the protein. The side-chains of the active site residues are shown.

Another homologous group that is potentially harmful to the host consists of hypothetical proteins CLIBASIA_00070 (gi: 254780135), CLIBASIA_04065 (gi: 254780914), CLIBASIA_04140 (gi: 254780929) and CLIBASIA_04540 (gi: 254781007). They are all predicted to harbor SPs. These four proteins share above 90% sequence identity with each other, so they likely preserve the same function. No confident homologs can be detected for them from organisms outside the *Candidatus* Liberibacter genus, indicating a high rate of evolution. The other bacterium in this genus, *Candidatus* Liberibacter solanacearum (*Ca.* L. solanacearum) has one copy of this unknown protein, and it is the pathogen that causes “zebra chip” disease in potatoes [75]. Due to the lack of detectable homologs that are studied experimentally, we cannot predict the structure or exact function of these proteins, but the fact that they are duplicated proteins with SPs, and that they are unique to two plant pathogens suggest their possible virulence roles.

Interestingly, 1% of the *Ca.* L. asiaticus proteins (listed in Table 2) lack detectable homologs (by BLAST or PSI-BLAST) from any other species (up to 05/22/2011, after the closely related *Ca.* L. solanacearum was sequenced). It is possible that these are “novel” genes originating in this bacterium, but it is more likely that they have diverged from their homologs so fast that the relationships are hardly detectable. Fast evolution is considered to be an important feature of virulence factors [76], and the presence of “redundant” and fast-evolving proteins in a highly reduced genome is consistent with pathogenic function. Moreover, many of these fast-evolving proteins are predicted to be either secreted or membrane-associated, further suggesting that they may be virulence factors associated with HLB.

#### Proteins with abnormal evolutionary history

We inspected the species associated with each protein’s closest homolog in the NR database detected by BLAST, since this information is indicative to the evolutionary history of a protein. We excluded the closest relative, *Ca.* L. solanacearum as some HGT events we are interested in might have happened before the divergence of Liberibacters. As expected, most (77%) *Ca.* L. asiaticus proteins have their closest homologs from *Alphaproteobacteria*. Only 11% have their closest homologs in other phylogenetic classes and the rest 12% lack detectable homologs outside the *Candidatus* Liberibacter genus (shown in Fig. 6).

Proteins whose closest homologs are from viruses are most likely related to bacteriophage integration. Most of them are from the recently integrated SC1 Liberibacter phage (colored green in Supplementary Table S6). It is important to note that some proteins from the integrated phage might be products of bacterial genes captured by the phage. In addition, our analysis revealed 13 phage-related proteins that do not belong to the SC1 Liberibacter phage (listed in Supplementary Table S6 and colored yellow). This indicates that another prophage might have integrated into *Ca.* L. asiaticus, but its genome likely has been reduced greatly over long time of evolution.

Out of these proteins with abnormal evolutionary history, we identified several potential virulence factors (colored red in Supplementary Table S6). One example is the hypothetical protein CLIBASIA_03975 (gi: 254780898). Homologous families of CLIBASLA_03975 detected by consensus of BLAST, RPS-BLAST and HHserach suggest its close relationship to the dual specificity phosphatase (DSP, protein serine/threonine and tyrosine phosphatase) protein family. Structure prediction also reveals strong similarity to phosphotyrosine protein phosphatases II fold proteins (shown in Fig. 7), with the functional motifs for DSP preserved and located in a shallow cleft on the surface of the structure. Protein Ser/Thr and Tyr kinase/phosphatases are typical components of eukaryotic signaling pathways. Although these phosphatases can participate in a bacterium’s own signaling pathway or adopt noncanonical function [77], they likely act as virulence factors since they can easily interact with the signaling system of the host [78], [79]. In addition, *Ca. L. asiaticus* seems to lack protein Ser/Thr or Tyr kinases that could function as counterparts of a DSP. This protein might participate in signaling pathways of a infected citrus plant, perhaps interfering with plant immune responses that involve protein kinase signaling cascades [80]. Most strikingly, a SP was predicted at the N-terminus of this protein, suggesting that it is secreted and further increasing the likelihood of its virulence role.

### Conclusions

We carried out computational analysis of all *Ca.* L. asiaticus proteins, and presented our results in a public website. With the assistance of our website, we performed manual curation to verify the results, predicted the subcellular localization, 3D structure and function of each protein and identified potential virulence factors. Our website serves as an encyclopedia of the *Ca.* L. asiaticus proteome to facilitate studies on the *Ca.* L. asiaticus proteins and contribute to the understanding of HLB.

## Supporting Information

Table S1 **Function predictions for all** ***Ca.*** **L. asiaticus proteins.** This table contains the following information, listed from the left to the right: (1) NCBI gi (sequence identifier in NCBI database), SEED id (sequence identifier in the SEED database), NCBI annotation, SEED annotation, curated function prediction (provided by us) and comments (additional comments about the protein, the transmembrane proteins and proteins with predicted signal peptides are marked).
(PDF)

Table S2 **Manual analysis of candidates for secreted proteins.**
(PDF)

Table S3 **Manual analysis of candidates for transmembrane proteins.**
(PDF)

Table S4 **Percentage of duplicated genes in proteomes of similar size to** ***Ca.*** **L. asiaticus.**
(PDF)

Table S5 **Homologous groups in** ***Ca.*** **L.asiaticus proteome.**
(PDF)

Table S6 ***Ca.*** **L. asiaticus Proteins with abnormal evolutionary history.**
(PDF)
