# Supplementary material for: Predictive Sequence Analysis of the Candidatus Liberibacter asiaticus Proteome
Source: PLoS One. 2012 Jul 18;7(7):e41071. doi: 10.1371/journal.pone.0041071 (PMC3399792; doi:10.1371/journal.pone.0041071)
Supplement: Table S2 — Manual analysis of candidates for secreted proteins. (PDF) [file pone.0041071.s002.pdf]

Table S2. Secreted protein candidates and results of manual analysis

GI	JUDGMENT	N1	N2	EVIDENCE
**254780384**	no SP, extracellular	0	0	1. Type I Secretion System substrate; 2. export by type I secretion system
**254780523**	No SP, extracellular	0	1	1. flagellar component; 2. do not have SP export by flagellar assembly machinery
**254780685**	No SP, outermembrane	0	0	1. flagellar component; 2. do not have SP export by flagellar assembly machinery
**254780380**	No SP, periplasmic	0	0	1. flagellar component; 2. do not have SP export by flagellar assembly machinery
**254780381**	No SP, periplasmic	0	0	1. flagellar component; 2. do not have SP export by flagellar assembly machinery
**254780379**	No SP, periplasmic	0	0	1. flagellar component; 2. do not have SP export by flagellar assembly machinery
**254780378**	No SP, extracellular	0	1	1. flagellar component; 2. do not have SP export by flagellar assembly machinery
**254780520**	No SP, extracellular	0	0	1. flagellar component; 2. do not have SP export by flagellar assembly machinery
**254780524**	No SP, extracellular	0	0	1. flagellar component; 2. do not have SP export by flagellar assembly machinery
**254780531**	No SP, extracellular	0	0	1. flagellar component; 2. do not have SP export by flagellar assembly machinery
**254780525**	No SP, extracellular	0	0	1. flagellar component; 2. do not have SP export by flagellar assembly machinery
**254780733**	No SP, periplasmic or extracellular	6	0	1. flp, pilus assembly protein; 2. has TMH but can be processed by TadV to become periplasmic. Do not have SP
**254780732**	No SP, periplasmic or extracellular	6	0	1. flp, pilus assembly protein; 2. has TMH but can be processed by TadV to become periplasmic. Do not have SP
**254780730**	No SP, periplasmic or extracellular	5	1	1. flp, pilus assembly protein; 2. has TMH but can be processed by TadV to become periplasmic. Do not have SP
**254780736**	No SP, periplasmic or extracellular	6	0	1. flp, pilus assembly protein; 2. has TMH but can be processed by TadV to become periplasmic. Do not have SP
**254780734**	No SP, periplasmic or extracellular	6	0	1. flp, pilus assembly protein; 2. has TMH but can be processed by TadV to become periplasmic. Do not have SP
**254781110**	No SP, periplasmic or extracellular	6	0	1. TadE or Tad F, pilus assembly protein; 2. has TMH but can be processed by TadV to become periplasmic. Do not have SP
**254780934**	No SP, periplasmic or extracellular	6	0	1. TadE or Tad F, pilus assembly protein; 2. has TMH but can be processed by TadV to become periplasmic. Do not have SP
**254780833**	No SP, periplasmic or extracellular	6	1	1. TadE or Tad F, pilus assembly protein; 2. has TMH but can be processed by TadV to become periplasmic. Do not have SP
**254780388**	No SP, periplasmic or extracellular	6	0	1. TadE or Tad F, pilus assembly protein; 2. has TMH but can be processed by TadV to become periplasmic. Do not have SP
**254781108**	No SP, periplasmic or extracellular	5	2	1. TadE or Tad F, pilus assembly protein; 2. has TMH but can be processed by TadV to become periplasmic. Do not have SP
**255764485**	SP, outermembrane	0	0	1. CpaD, pilus assembly; 2. orthologs proved by Phobius and one mode of SignalP; 3. start point is wrong, should remove "MMVEYMITILFGGVCFKGLANMRSL ISCLKTIFWKNFFLRTL", then Phobius and one mode of SignalP predict SP
**254780707**	SP, extracytoplasmic	3	1	1. Function; 2. ortholog proved by both modes of SignalP and Phobius; 3. COG suggest this protein is usually periplasmic
**254780396**	SP, extracytoplasmic	6	1	1. function; 2. ortholog proved by both modes of SignalP and Phobius; 3. pfam suggest this protein contains domain that is usually periplasmic
**254780698**	SP, extracytoplasmic	4	1	1. function; 2. pfam suggest this protein contains domain that is usually periplasmic; 3. start point wrong, should remove "MLQQKIQSSAKKTFLTYPYGSIYIQRNIQL", then 4 program predict SPs
**254780930**	SP, extracytoplasmic	3	1	1. function; 2. ortholog proved by both modes of SignalP and Phobius
**254780700**	SP, extracytoplasmic?	6	1	1. ortholog proved by both modes of SignalP and Phobius;<br>2. COG suggest this protein is usually periplasmic
**254780772**	SP, extracytoplasmic?	6	1	1. function; 2. start point is wrong, should remove "MHKSTEDFRRIKRLLEKYFPRSFQ", then phobius will also predict SP
**254781112**	SP, extracytoplasmic?	6	1	1. function; 2. ortholog can be verified by both modes of SignalP and Phobius
**254780764**	SP, extracytoplasmic?	6	1	1. function; 2. Ortholog; 3. start point is wrong, should remove "MFTHAEKILYS LDLRKY", then both phibus and signalP predict it as signal protien
**254781174**	SP, extracytoplasmic?	6	1	1. function; 2. ortholog
**254780561**	SP, extracytoplasmic	4	2	Function
**254780314**	SP, extracytoplasmic	0	2	1. function; 2. ortholog proved by two modes of SignalP and Phobius
**254780635**	SP, extracytoplasmic	0	2	ortholog proved by one mode of SignalP and Phobius
**254780350**	SP, extracytoplasmic	4	2	ortholog proved by one mode of SignalP and Phobius, at least periplasm
**254780953**	SP, extracytoplasmic	0	2	1. function; 2. ortholog proved by one mode of SignalP and Phobius
**254780898**	SP, extracytoplasmic	6	2	1. function; 2. ortholog proved by one mode of SignalP and MEMSAT
**254780395**	SP, extracytoplasmic	2	2	1. function; 2. ortholog proved by two modes of SignalP and Phobius
**254780798**	SP, extracytoplasmic	5	2	1. function; 2. ortholog confirmed by two modes of SignalP
**254780728**	SP, extracytoplasmic	5	2	Function
**254780592**	SP, extracytoplasmic	4	2	Function
**254780376**	SP, extracytoplasmic	2	2	1. function, although it is Flagellar assembly protein, but this one is probably transported by Sec into periplasm to set up the flagellar machinery, it is FlgI, and it is

				an exception; 2. ortholog, confirmed by two modes of signalP too
**254780477**	SP, extracytoplasmic	5	2	ortholog proved by one mode of SignalP and Phobius
**254780745**	SP, extracytoplasmic	3	2	1. function; 2. pfam suggest this protein contains domain that is usually periplasmic
**254781221**	SP, extracytoplasmic	0	2	ortholog predicted to have SP
**254781045**	SP, extracytoplasmic	0	2	1. function; 2. ortholog, confirmed by two modes of signalP and phobius too
**254780221**	SP, extracytoplasmic	0	3	
**254780528**	SP, extracytoplasmic	0	3	Function
**254780959**	SP, extracytoplasmic	5	3	
**254780954**	SP, extracytoplasmic	5	3	
**254781078**	SP, extracytoplasmic	4	3	
**254780556**	SP, extracytoplasmic	2	3	
**254780906**	SP, extracytoplasmic	0	3	
**254780737**	SP, extracytoplasmic	0	3	
**254780735**	SP, extracytoplasmic	4	3	a bit weird, as it is flp homolog
**254780281**	SP, extracytoplasmic	5	3	Ortholog
**254780342**	SP, extracytoplasmic	2	3	Function
**254780984**	SP, extracytoplasmic	0	3	Close homolog is 254780981, which is predicted to have SP by 2 methods out of 4
**254780935**	SP, extracytoplasmic	4	3	Function
**254780547**	SP, extracytoplasmic	2	3	Function
**254781014**	SP, extracytoplasmic	4	3	
**254781128**	SP, extracytoplasmic	3	3	
**254780759**	SP, extracytoplasmic	0	3	
**254781010**	SP, extracytoplasmic	0	3	
**254780377**	SP, extracytoplasmic	3	3	function, although it is Flagellar assembly protein, but this one is probably transported by Sec into periplasm, it is FlgA, and it is an exception
**254780375**	SP, extracytoplasmic	0	3	
**254780374**	SP, extracytoplasmic	2	3	function, although it is Flagellar assembly protein, but this one is probably transported by Sec into periplasm, it is FlgH, and it is an exception
**254780717**	SP, extracytoplasmic	4	3	function
**254780435**	SP, extracytoplasmic	3	3	function
**254780965**	SP, extracytoplasmic	5	3	
**254780747**	SP, extracytoplasmic	6	3	1. Function; 2. structure prediction, the TMH predicted in the middle of the protein is not true judging from the structure template
**254780207**	SP, extracytoplasmic	0	3	
**254780141**	SP, extracytoplasmic	3	3	function
**254780589**	SP, extracytoplasmic	2	3	
**254780563**	SP, extracytoplasmic	0	4	function
**254780951**	SP, extracytoplasmic	3	4	
**254780170**	SP, extracytoplasmic	4	4	function
**254780308**	SP, extracytoplasmic	4	4	function
**254780907**	SP, extracytoplasmic	0	4	
**254780909**	SP, extracytoplasmic	0	4	
**254781156**	SP, extracytoplasmic	0	4	
**254781157**	SP, extracytoplasmic	0	4	
**254780607**	SP, extracytoplasmic	0	4	
**254780199**	SP, extracytoplasmic	5	4	function
**254781207**	SP, extracytoplasmic	5	4	ortholog
**254780542**	SP, extracytoplasmic	0	4	
**254780548**	SP, extracytoplasmic	5	4	function
**254781121**	SP, extracytoplasmic	0	4	
**254780209**	SP, extracytoplasmic	3	4	
**254780727**	SP, extracytoplasmic	3	4	function
**254780598**	SP, extracytoplasmic	0	4	function
**254780844**	SP, extracytoplasmic	4	4	
**254780537**	SP, extracytoplasmic	2	4	function
**254781003**	SP, extracytoplasmic	3	4	
**254780443**	SP, extracytoplasmic	4	4	
**254780981**	SP, extracytoplasmic	1	2	Close homolog is 254780984, which is predicted to have SP by 3 methods out of 4
**254780220**	SP, extracytoplasmic	0	2	
**255764500**	SP, extracytoplasmic	2	2	
**254781159**	SP, extracytoplasmic	0	2	
**254781005**	SP, extracytoplasmic	0	2	
**254780886**	SP, extracytoplasmic	3	2	
**254780963**	SP, extracytoplasmic	3	2	
**254780914**	SP, extracytoplasmic?	6	2	
**254780135**	SP, extracytoplasmic?	6	2	

**254780929**	SP, extracytoplasmic?	6	2	
**254781007**	SP, extracytoplasmic?	6	2	
**254780980**	SP, extracytoplasmic	0	1	close homolog is 254780556, which is predicted to have SP by 3 out of 4 methods
**254780311**	cytoplasmic	0	1	1. funciton as cytoplasmic enzyme; 2. structure prediction
**254780313**	cytoplasmic	0	1	1. funciton, work with ribosome; 2. structure prediction
**254780315**	cytoplasmic	0	1	
**254781149**	cytoplasmic	0	1	Function
**254781058**	cytoplasmic	0	1	1. function, this is the C-terminal part of phage lysozyme; 2. structure prediction
**254781197**	cytoplasmic	0	1	Function
**254781071**	cytoplasmic	0	1	1. this is just the middle piece of a protein; 2. structure prediction
**254780392**	cytoplasmic	0	1	1. Function; 2. structure prediction
**254780482**	cytoplasmic	0	1	1. function; 2. structure prediction
**255764495**	cytoplasmic	0	1	ortholog cannot be verified by either mode of signalP or Phobius
**255764462**	cytoplasmic	0	1	function
**254780908**	cytoplasmic	0	1	
**254780918**	cytoplasmic	0	1	
**254780912**	cytoplasmic	0	1	
**254781026**	cytoplasmic	0	1	1. function; 2. this is the middle part of a big protein; 3. structure prediction
**254780195**	cytoplasmic	2	1	1. function; 2. structure prediction
**254780942**	cytoplasmic	0	1	function
**254781205**	cytoplasmic	0	1	
**254781162**	cytoplasmic	0	1	function
**254780168**	cytoplasmic	0	1	1. function; 2. structure prediction;<br>3. ortholog proved by one mode of SignalP and Phobius
**254780164**	cytoplasmic	0	1	Function
**254780768**	cytoplasmic	0	1	1. Ortholog; 2. pfam
**254780640**	cytoplasmic	0	1	Function
**254780543**	cytoplasmic	0	1	Function
**254780227**	cytoplasmic	3	1	function as metabolic enzyme
**254780724**	cytoplasmic	0	1	Function
**254780470**	cytoplasmic	0	1	1. function; 2. structure prediction
**254780920**	cytoplasmic	2	1	Function
**peg_1087**	cytoplasmic	0	1	
**254780132**	cytoplasmic	0	1	Function
**254780156**	cytoplasmic	0	1	Function
**254781097**	cytoplasmic	5	1	1. structure prediction; 2. tiger say this step occur in cytoplasm
**254780154**	cytoplasmic	0	1	
**254781099**	cytoplasmic	0	1	1. structure prediction; 2. ortholog can only be verified by Phobius;<br>3. PRK says this step is cytoplasmic
**254780787**	cytoplasmic	0	1	function, translation initiation factor IF-2
**254781036**	cytoplasmic	0	1	1. function; 2. pfam
**254781031**	cytoplasmic	2	1	1. Function; 2. structure prediction
**254780439**	cytoplasmic	2	1	1. function; 2. pfam
**254780434**	cytoplasmic	0	1	1. function; 2. pfam
**peg_473**	cytoplasmic	0	1	function
**254780322**	cytoplasmic	0	1	1. function; 2. structure prediction
**254780742**	cytoplasmic	0	1	1. function; 2. pfam
**254780219**	cytoplasmic	0	1	
**254780261**	cytoplasmic	0	1	function, ribosome protein
**254780266**	cytoplasmic	0	1	function, ribosome protein
**254780361**	cytoplasmic	0	1	function, ribosome protein
**254780687**	cytoplasmic	0	1	Function, flagellar cytoplasmic component
**254780682**	cytoplasmic?	0	1	
**254780510**	cytoplasmic?	0	1	
**254781039**	cytoplasmic	3	2	function as metabolic enzyme
**peg_887**	cytoplasmic	0	2	function in tRNA processing
**254781015**	cytoplasmic	0	2	this does not look like a complete protein, ortholog is a Multi-TMHs protein, but this one lost most of the transmembrane part.
**peg_1064**	cytoplasmic	0	2	ortholog not predicted to have SP by both modes of SignalP and Phobius
**254781048**	cytoplasmic	3	2	fuction in TCA cycle
**254780319**	cytoplasmic	0	3	function, ribosome protein
**255764476**	transmembrane, SP not likely real	6	1	1. function; 2. multi-TMHs
**255764499**	transmembrane	6	1	1. Single-TMH, major part cytoplasmic<br>2. ortholog cannot be verified by either mode of signalP or phobius
**254780140**	Transmembrane, SP not likely true	6	1	1. single-TMH, major part periplasmic<br>2. ortholog can be proved by only one mode of signalp

**254780519**	transmembrane, SP not likely real	6	1	1. Function, flagellar transmembrane component; 2. multi-TMHs
**254780695**	transmembrane, SP not likely real	6	1	1. Function, flagellar transmembrane component; 2. double-TMH
**255764488**	transmembrane, SP not likely real	6	1	1. Function, flagellar transmembrane component; 2. double-TMH
**254780507**	transmembrane, SP not likely true	6	1	1. Function, flagellar transmembrane component;<br>2. Single-TMH, major part cytoplasmic
**254780545**	transmembrane, but SP not likely real	6	1	Single-TMH, major part cytoplasmic
**254780752**	transmembrane, SP not likely real	6	1	1. single-TMH, major part periplasmic; 2. ortholog
**254780577**	transmembrane, SP not likely real	6	1	1. function; 2. single-TMH, major part periplasmic
**254780623**	transmembrane, SP not likely real	6	1	1. Single-TMH at C-terminal, major part periplasmic; 2. ortholog
**254780436**	transmembrane, SP not likely real	4	1	single-TMH, major part periplasmic
**254780961**	transmembrane, SP not likely real?	6	1	1. single-TMH, major part periplasmic; 2. ortholog do not have SP by neither mode of SignalP or Phobius; 3. start point is wrong, should add "MSFNVRNFVLWI" in front, and it has one SP, one TMH
**255764508**	transmembrane, SP might be right	6	1	double-TMH
**254780367**	transmembrane, SP not likely real	6	1	multi-TMHs
**254780866**	transmembrane, SP not likely real	6	1	multi-TMHs
**254780568**	transmembrane, SP not likely real	6	1	multi-TMHs
**255764466**	transmembrane, SP not likely real	6	1	multi-TMHs
**254780402**	transmembrane, SP not likely real	6	1	double-TMH
**254780228**	transmembrane, SP not likely real	6	1	1. double-TMHs; 2. start wrong, should add "MMLILFI", then it has double TMHs
**254780852**	transmembrane, SP not likely real	6	1	multi-TMHs
**254780917**	transmembrane, SP not likely real	6	1	1. function; 2. multi-TMHs
**254780708**	transmembrane, SP not likely real	6	1	multi-TMHs
**254780705**	transmembrane, SP not likely real	6	1	1. function; 2. multi-TMHs
**254781033**	transmembrane, SP not likely real	6	1	multi-TMHs
**254780450**	transmembrane, SP not likely real	3	1	double-TMH
**254780774**	transmembrane, SP not likely real	6	1	multi-TMHs
**254781079**	transmembrane, SP not likely real	5	1	multi-TMHs
**254780793**	transmembrane, SP not likely real	6	1	multi-TMHs
**254780171**	transmembrane, SP not likely real	6	1	1. function; 2. multi-TMHs
**254780483**	transmembrane, SP not likely real	6	1	1. function; 2. multi-TMHs
**255764497**	transmembrane, SP not likely real	6	1	multi-TMHs
**255764468**	transmembrane, SP not likely real	6	1	1. multi-TMHs; 2. start point is wrong, should add "MSRKVRKFDTVFPFTNYI RYSIISLPVVFLGVVCCLLAIILIIFKDSLDNSISFGLFG" in front
**254780860**	transmembrane, SP not likely real	6	1	multi-TMHs
**255764512**	transmembrane, SP not likely real	6	1	1. Single-TMH, major part cytoplasmic<br>2. ortholog cannot be verified by either mode of signalP or phobius
**254781169**	transmembrane, SP not likely real	6	1	multi-TMHs
**254780799**	transmembrane, SP not likely real	6	1	multi-TMHs
**254780425**	transmembrane, SP not likely real	6	1	Multi-TMHs
**254780494**	transmembrane, SP not likely real	6	1	1. function; 2. Multi-TMHs
**254780335**	transmembrane, SP not likely real	6	1	Multi-TMHs
**254780721**	transmembrane, SP not likely real	6	1	Multi-TMHs
**254780298**	transmembrane, SP not likely real	6	1	1. function; 2. Multi-TMHs
**254780291**	transmembrane, SP not likely real	6	1	1. Single-TMH, major part periplasmic<br>2. ortholog predicted to have SP by only one mode of SignalP
**254780593**	transmembrane, SP not likely real	6	1	Multi-TMHs
**254780576**	transmembrane, SP not likely real	6	1	1. function; 2. Multi-TMHs
**254780841**	transmembrane, SP not likely real	6	1	Multi-TMHs
**254780924**	transmembrane, SP not likely real	6	1	Multi-TMHs
**254780925**	transmembrane, SP not likely real	6	1	1. function; 2. Multi-TMHs
**254781178**	transmembrane, SP not likely real	6	1	1. Multi-TMHs; 2. Ortholog
**254780719**	transmembrane, SP not likely real	6	1	1. function; 2. Multi-TMHs
**254781002**	transmembrane, SP not likely real	6	1	Multi-TMHs
**254780323**	transmembrane, SP not likely real	5	1	1. function; 2. multi-TMHs
**254780967**	transmembrane, SP not likely real	6	1	Multi-TMHs
**254780815**	transmembrane, SP not likely real	5	1	1. Single-TMH, major part cytoplasmic; 2. Pfam;<br>3. ortholog predicted to have SP only by Phobius
**254780749**	transmembrane, SP not likely real	6	1	Multi-TMHs
**254781189**	transmembrane, SP not likely real	6	1	Multi-TMHs
**254781042**	transmembrane, SP not likely real	6	1	Multi-TMHs
**254780558**	transmembrane, SP not likely real	6	1	1. function; 2. Pfam; 3.single TMH at N-terminus and mainly periplasmic,
**254781158**	transmembrane, SP not likely real	6	1	1. single TMH at N-terminus and mainly periplasmic;<br>2.ortholog has SP by Phobius and one mode of SignalP
**254780421**	transmembrane, SP not likely real	6	1	single TMH at N-terminus and mainly periplasmic,
**254780386**	transmembrane, SP not likely real	5	1	single TMH at N-terminus and mainly periplasmic,

**254780572**	transmembrane, SP not likely real	6	1	1. Function, pilus transmembrane component; 2. single-TMH, major part periplasmic
**254780679**	transmembrane, SP not likely real	6	2	might be single transmembrane protein, and the C-terminal is cytoplasmic
**254780671**	transmembrane, SP not likely real	6	2	1. function; 2. might be single-TMH after cleaving SP, major part is cytoplasmic
**254780532**	transmembrane, SP not likely real	5	2	1. single-TMH, major part periplasmic;<br>2. ortholog can be proved by only one mode of signalp
**peg_237**	transmembrane, SP not likely real	6	2	single-TMH in the middle
**peg_789**	transmembrane, SP not likely real	6	2	multi-TMHs
**254781098**	transmembrane, SP not likely real	6	2	Multi-TMHs
**254780896**	transmembrane, but SP is probably real	6	2	1. function; 2. multi-TMHs
**254780372**	transmembrane, but SP is probably real	6	3	1. function, flagellar transmembrane component; 2. Multi-TMHs
**254780729**	trasmembrane, but SP might be true	6	3	1. function; 2. Multi-TMHs
**254780196**	trasmembrane, but SP might be true	6	3	1. function; 2. after cleaveage of SP, single-TMH, major cytoplasmic
**254780468**	transmembrane, but SP is probably real	6	4	Multi-TMHs

N1: number of methods (out of 6) that predict the protein to have transmembrane helix (helices)
N2: number of methods (out of 4) that predict the protein to have a signal peptide
SP: signal peptide
TMH: transmembrane helix

Cell color:
Yellow: extracytoplasmic proteins that are without signal peptides and that are secreted in Sec-independent pathways;
Light green: proteins with signal peptides and they are likely function in the extracytoplasmic space;
Pink: cytoplasmic proteins
Light blue: transmembrane proteins that are located in the inner membrane of this Gram-negative bacterium.
