# Supplementary material for: Predictive Sequence Analysis of the Candidatus Liberibacter asiaticus Proteome
Source: PLoS One. 2012 Jul 18;7(7):e41071. doi: 10.1371/journal.pone.0041071 (PMC3399792; doi:10.1371/journal.pone.0041071)
Supplement: Table S3 — Manual analysis of candidates for transmembrane proteins. (PDF) [file pone.0041071.s003.pdf]

Table S3. Transmembrane protein candidates and results of manual analysis

GI	Prediction	N	Evidence
254780811	transmembrane	6	1. cog; 2. multi-TMHs
254780551	transmembrane	6	1. funciton; 2. multi-TMHs
254780539	transmembrane	6	1. funciton; 2. multi-TMHs; 3. structure prediction
254780843	transmembrane	6	1. funciton; 2. multi-TMHs; 3. structure prediction
254780864	transmembrane	6	1. funciton; 2. double-TMHs
254780867	transmembrane	6	1. funciton; 2. multi-TMHs
254780903	transmembrane	6	1. funciton; 2. double-TMHs
254781029	transmembrane	6	1. funciton; 2. double-TMHs
254781103	transmembrane	6	1. funciton;<br>2. single-TMH close to N-terminus major part periplasmic
254781184	transmembrane	6	1. funciton;<br>2. single-TMH close to N-terminus major part cytoplasmic
254780690	transmembrane	6	function
254780512	transmembrane	6	function
254780138	transmembrane	6	1. funciton; 2. multi-TMHs; 3. structure prediction
254780149	transmembrane	6	1. funciton; 2. structure prediction; 3. single-TMH in the middle
254780172	transmembrane	6	1. funciton; 2. multi-TMHs; 3. structure prediction
254780193	transmembrane	6	1. funciton; 2. multi-TMHs; 3. structure prediction
254780205	transmembrane	6	1. funciton; 2. multi-TMHs
254780223	transmembrane	6	1. funciton; 2. multi-TMHs; 3. structure prediction
254780230	transmembrane	6	1. funciton; 2. double-TMHs
254780241	transmembrane	6	1. funciton; 2. multi-TMHs; 3. structure prediction
254780302	transmembrane	6	1. funciton; 2. multi-TMHs
254780341	transmembrane	6	1. funciton; 2. multi-TMHs; 3. structure prediction
254780360	transmembrane	6	1. funciton; 2. multi-TMHs
255764465	transmembrane	6	1. funciton; 2. multi-TMHs; 3. structure prediction
254780373	transmembrane	6	1. function;<br>2. single-TMH close to N-terminus major part periplasmic
255764467	transmembrane	6	1. funciton; 2. multi-TMHs; 3. structure prediction
254780387	transmembrane	6	1. funciton; 2. multi-TMHs; 3. structure prediction
255764469	transmembrane	6	1. funciton; 2. multi-TMHs
254780411	transmembrane	6	1. funciton; 2. multi-TMHs
255764470	transmembrane	6	1. funciton; 2. multi-TMHs
254780452	transmembrane	6	1. funciton; 2. multi-TMHs
254780463	transmembrane	6	1. function; 2. single-TMH at C-terminus major part cytoplasmic
254780471	transmembrane	6	1. funciton; 2. multi-TMHs
254780472	transmembrane	6	1. funciton; 2. multi-TMHs; 3. structure prediction
254780473	transmembrane	6	1. funciton; 2. multi-TMHs; 3. structure prediction
254780773	transmembrane	6	1. funciton; 2. multi-TMHs
254780746	transmembrane	6	1. function;<br>2. single-TMH close to N-terminus major part periplasmic
254780722	transmembrane	6	1. function; 2. double-TM
255764486	transmembrane	6	1. funciton; 2. multi-TMHs; 3. structure prediction
254780701	transmembrane	6	1. funciton; 2. multi-TMHs; 3. structure prediction
255764501	transmembrane	6	1. funciton; 2. multi-TMHs; 3. structure prediction
254780549	transmembrane	6	1. function; 2. pfam and tiger suggests this is single TM protein leaving major part periplasmic
254780540	transmembrane	6	1. funciton; 2. multi-TMHs; 3. structure prediction
254780832	transmembrane	6	1. funciton; 2. multi-TMHs; 3. structure prediction
254780862	transmembrane	6	1. funciton; 2. multi-TMHs
254780865	transmembrane	6	1. funciton; 2. multi-TMHs
254780939	transmembrane	6	1. function;

			2. single-TMH close to N-terminus major part periplasmic
254780982	transmembrane	6	1. funciton; 2. multi-TMHs
254780993	transmembrane	6	1. funciton; 2. multi-TMHs
254781041	transmembrane	6	1. funciton; 2. multi-TMHs; 3. structure prediction
254781085	transmembrane	6	1. funciton; 2. structure prediction;<br>3. single-TMH close to N-terminus major part cytoplasmic
254781086	transmembrane	6	1. funciton; 2. structure prediction;<br>3. single-TMH close to N-terminus major part cytoplasmic
254781087	transmembrane	6	1. funciton; 2. double-TM; 3. structure prediction
254781088	transmembrane	6	1. funciton; 2. multi-TMHs; 3. structure prediction
254781100	transmembrane	6	1. funciton; 2. multi-TMHs
254781113	transmembrane	6	1. funciton; 2. multi-TMHs; 3. structure prediction
254781171	transmembrane	6	1. funciton; 2. multi-TMHs
254780343	transmembrane	6	1. funciton; 2. multi-TMHs; 3. structure prediction
254780697	transmembrane	6	1. function; 2. double-TM
254780571	transmembrane	6	1. function TadG is membrane protein;<br>2. single-TMH close to N-terminus major part periplasmic
254780803	transmembrane	6	1. pfam; 2. cog;<br>3. single-TMH close to N-terminus major part periplasmic
254780695	transmembrane	6	
255764488	transmembrane	6	
255764508	transmembrane	6	
254780402	transmembrane	6	
254780372	transmembrane	6	
254780519	transmembrane	6	
254780511	transmembrane	6	
254780468	transmembrane	6	
254780729	transmembrane	6	
peg_789	transmembrane	6	
254781098	transmembrane	6	
254780896	transmembrane	6	
254780171	transmembrane	6	
254780228	transmembrane	6	
254780298	transmembrane	6	
254780335	transmembrane	6	
254780367	transmembrane	6	
254780425	transmembrane	6	
254780483	transmembrane	6	
254780494	transmembrane	6	
254780793	transmembrane	6	
254780799	transmembrane	6	
254780841	transmembrane	6	
254780852	transmembrane	6	
254780860	transmembrane	6	
254780866	transmembrane	6	
254780917	transmembrane	6	
254780924	transmembrane	6	
254780925	transmembrane	6	
254780967	transmembrane	6	
254781002	transmembrane	6	
254781033	transmembrane	6	
254781042	transmembrane	6	
254781169	transmembrane	6	
254781178	transmembrane	6	

254781189	transmembrane	6	
255764466	transmembrane	6	
255764468	transmembrane	6	
255764497	transmembrane	6	
254780568	transmembrane	6	
254780576	transmembrane	6	
254780593	transmembrane	6	
254780705	transmembrane	6	
254780708	transmembrane	6	
254780719	transmembrane	6	
254780721	transmembrane	6	
254780749	transmembrane	6	
254780774	transmembrane	6	
255764476	transmembrane	6	
peg_237	transmembrane	6	
254780623	transmembrane	6	
254780572	transmembrane	6	
254780529	transmembrane	6	
254780752	transmembrane	6	
254781158	transmembrane	6	
254780577	transmembrane	6	
254780421	transmembrane	6	
254780558	transmembrane	6	
254780291	transmembrane	6	
254780140	transmembrane	6	
254780961	transmembrane	6	
255764499	transmembrane	6	
254780507	transmembrane	6	
254780545	transmembrane	6	
255764512	transmembrane	6	
254780196	transmembrane	6	
254780671	transmembrane	6	
254780679	transmembrane	6	
254780159	transmembrane	6	double-TM
254780179	transmembrane	6	single-TMH at C-terminus major part cytoplasmic
254780198	transmembrane	6	double-TM
254780204	transmembrane	6	multi-TMHs
254780235	transmembrane	6	single-TMH at C-terminus major part cytoplasmic
254780284	transmembrane	6	single-TMH in the middle
254780324	transmembrane	6	single-TMH in the middle
254780354	transmembrane	6	single-TMH in the middle
254780790	transmembrane	6	1. structure prediction; 2. multi-TMHs
peg_476	transmembrane	6	1. structure prediction; 2. multi-TMHs
peg_477	transmembrane	6	1. structure prediction; 2. multi-TMHs
254780612	transmembrane	6	double-TM
254780600	transmembrane	6	multi-TMHs
254780579	transmembrane	6	single-TMH close to N-terminus major part cytoplasmic
254780578	transmembrane	6	multi-TMHs
254780575	transmembrane	6	1. structure prediction; 2. multi-TMHs
255764502	transmembrane	6	single-TMH in the middle
255764503	transmembrane	6	single-TMH in the middle
254780863	transmembrane	6	single-TMH in the middle
254780878	transmembrane	6	single-TMH in the middle

254780879	transmembrane	6	single-TMH close to N-terminus major part periplasmic
254780944	transmembrane	6	double-TM
254780986	transmembrane	6	double-TM
254780988	transmembrane	6	double-TM
254781013	transmembrane	6	double-TM
254781022	transmembrane	6	multi-TMHs
254781034	transmembrane	6	single-TMH close to N-terminus major part periplasmic
254781075	transmembrane	6	double-TM
254781104	transmembrane	6	single-TMH close to N-terminus major part cytoplasmic
254781109	transmembrane	6	multi-TMHs
254781163	transmembrane	6	double-TM
254781199	transmembrane	6	double-TM
peg_1140	transmembrane	6	single-TMH close to C-terminus major part cytoplasmic
254781224	transmembrane	6	single-TMH close to C-terminus major part cytoplasmic
254780338	transmembrane	6	1. structure prediction;<br>2. double-TM according to structure model
254780356	transmembrane	6	multi-TMHs
254780357	transmembrane	6	multi-TMHs
254780565	transmembrane	6	single-TMH close to C-terminus major part cytoplasmic
254780723	transmembrane	6	double-TM
254780401	transmembrane	5	1. funciton; 2. structure prediction;<br>3. single-TMH close to N-terminus major part periplasmic
254780474	transmembrane	5	1. funciton; 2. structure prediction;<br>3. double-TM according to structure model
254780958	transmembrane	5	1. function;<br>2. single-TMH close to N-terminus major part periplasmic
254780323	transmembrane	5	
254781079	transmembrane	5	
254780386	transmembrane	5	
254780532	transmembrane	5	
254780815	transmembrane	5	
254780448	transmembrane	5	single-TMH close to N-terminus major part periplasmic
254781181	transmembrane	5	single-TMH close to N-terminus major part cytoplasmic
254781119	transmembrane	5	single-TMH close to C-terminus
254781198	transmembrane	5	single-TMH close to C-terminus major part cytoplasmic
254780500	transmembrane	5	single-TMH close to N-terminus major part periplasmic
peg_788	transmembrane	4	1. function;<br>2. single-TMH close to N-terminus major part cytoplasmic
254780436	transmembrane	4	
254780450	transmembrane	3	
peg_804	periplasmic or extracellular	4	signal peptide
255764485	outermembrane, with SP	5	1. signal peptide;<br>2. start point is wrong, should remove "MMVEYMITILFGGV CFKGLANMRSLISCLKTIFWKNFFLRTL", then Phobius and one mode of SignalP predict this protein with SP
254780396	extracytoplasmic, with SP	6	signal peptide
254780747	extracytoplasmic, with SP	6	signal peptide
254780898	extracytoplasmic, with SP	6	signal peptide
254780135	extracytoplasmic, with SP	6	signal peptide
254781007	extracytoplasmic, with SP	6	signal peptide
254780929	extracytoplasmic, with SP	6	signal peptide
254780914	extracytoplasmic, with SP	6	signal peptide
254780764	extracytoplasmic, with SP	6	signal peptide
254780700	extracytoplasmic, with SP	6	signal peptide

254780772	extracytoplasmic, with SP	6	signal peptide
254781112	extracytoplasmic, with SP	6	signal peptide
254781174	extracytoplasmic, with SP	6	signal peptide
254781207	extracytoplasmic, with SP	5	signal peptide
254780548	extracytoplasmic, with SP	5	signal peptide
254780199	extracytoplasmic, with SP	5	signal peptide
254780965	extracytoplasmic, with SP	5	signal peptide
254780959	extracytoplasmic, with SP	5	signal peptide
254780954	extracytoplasmic, with SP	5	signal peptide
254780281	extracytoplasmic, with SP	5	signal peptide
254780798	extracytoplasmic, with SP	5	signal peptide
254780728	extracytoplasmic, with SP	5	signal peptide
254780477	extracytoplasmic, with SP	5	signal peptide
254780844	extracytoplasmic, with SP	4	signal peptide
254780443	extracytoplasmic, with SP	4	signal peptide
254780308	extracytoplasmic, with SP	4	signal peptide
254780170	extracytoplasmic, with SP	4	signal peptide
254781078	extracytoplasmic, with SP	4	signal peptide
254781014	extracytoplasmic, with SP	4	signal peptide
254780935	extracytoplasmic, with SP	4	signal peptide
254780735	extracytoplasmic, with SP	4	signal peptide
254780717	extracytoplasmic, with SP	4	signal peptide
254780698	extracytoplasmic, with SP	4	signal peptide
254780350	extracytoplasmic, with SP	4	signal peptide
254780592	extracytoplasmic, with SP	4	signal peptide
254780561	extracytoplasmic, with SP	4	signal peptide
254781003	extracytoplasmic, with SP	3	signal peptide
254780951	extracytoplasmic, with SP	3	signal peptide
254780727	extracytoplasmic, with SP	3	signal peptide
254780209	extracytoplasmic, with SP	3	signal peptide
254781128	extracytoplasmic, with SP	3	signal peptide
254780435	extracytoplasmic, with SP	3	signal peptide
254780377	extracytoplasmic, with SP	3	signal peptide
254780141	extracytoplasmic, with SP	3	signal peptide
254780745	extracytoplasmic, with SP	3	signal peptide
254780963	extracytoplasmic, with SP	3	signal peptide
254780886	extracytoplasmic, with SP	3	signal peptide
254780707	extracytoplasmic, with SP	3	signal peptide
254780930	extracytoplasmic, with SP	3	signal peptide
254780537	extracytoplasmic, with SP	2	signal peptide
254780589	extracytoplasmic, with SP	2	signal peptide
254780556	extracytoplasmic, with SP	2	signal peptide
254780547	extracytoplasmic, with SP	2	signal peptide
254780376	extracytoplasmic, with SP	2	signal peptide
254780374	extracytoplasmic, with SP	2	signal peptide
254780342	extracytoplasmic, with SP	2	signal peptide
254780395	extracytoplasmic, with SP	2	signal peptide
255764500	extracytoplasmic, with SP	2	signal peptide
254780375	extracytoplasmic, with SP	2	signal peptide
254780737	extracytoplasmic, with SP	2	signal peptide
254780635	extracytoplasmic, with SP	2	signal peptide
254780607	extracytoplasmic, with SP	2	signal peptide
254780980	extracytoplasmic, with SP	2	signal peptide

254781010	extracytoplasmic, with SP	2	signal peptide
254781121	extracytoplasmic, with SP	2	signal peptide
254781156	extracytoplasmic, with SP	2	signal peptide
254781159	extracytoplasmic, with SP	2	signal peptide
254780314	extracytoplasmic, with SP	1	signal peptide
254780759	extracytoplasmic, with SP	1	signal peptide
254780598	extracytoplasmic, with SP	1	signal peptide
254780563	extracytoplasmic, with SP	1	signal peptide
254780542	extracytoplasmic, with SP	1	signal peptide
254780528	extracytoplasmic, with SP	1	signal peptide
254780906	extracytoplasmic, with SP	1	signal peptide
254780907	extracytoplasmic, with SP	1	signal peptide
254780909	extracytoplasmic, with SP	1	signal peptide
254780953	extracytoplasmic, with SP	1	signal peptide
254780981	extracytoplasmic, with SP	1	signal peptide
254781005	extracytoplasmic, with SP	1	signal peptide
254781045	extracytoplasmic, with SP	1	signal peptide
254781157	extracytoplasmic, with SP	1	signal peptide
254781221	extracytoplasmic, with SP	1	signal peptide
254780525	extracytoplasmic, withitout SP	1	1. flagellar component;<br>2. do not have SP export by flagellar assembly machinery
254780388	extracytoplasmic, TM will be cleaved	6	periplasmic can be processed by TadV but to SP
254780833	extracytoplasmic, TM will be cleaved	6	periplasmic can be processed by TadV but to SP
254780934	extracytoplasmic, TM will be cleaved	6	periplasmic can be processed by TadV but to SP
254781110	extracytoplasmic, TM will be cleaved	6	periplasmic can be processed by TadV but to SP
254780732	extracytoplasmic, TM will be cleaved	6	periplasmic can be processed by TadV but to SP
254780733	extracytoplasmic, TM will be cleaved	6	periplasmic can be processed by TadV but to SP
254780734	extracytoplasmic, TM will be cleaved	6	periplasmic can be processed by TadV but to SP
254780736	extracytoplasmic, TM will be cleaved	6	periplasmic can be processed by TadV but to SP
254781108	extracytoplasmic, TM will be cleaved	5	periplasmic can be processed by TadV but to SP
254780730	extracytoplasmic, TM will be cleaved	5	periplasmic can be processed by TadV but to SP
254780315	cytoplasmic?	2	maybe not real protein
254781134	cytoplasmic?	2	
254781203	cytoplasmic?	2	
254781097	cytoplasmic	5	function
254780740	cytoplasmic	4	1. function; 2. structure prediction
254780901	cytoplasmic	4	1. function; 2. structure prediction
254780933	cytoplasmic	4	1. function; 2. structure prediction
254781018	cytoplasmic	4	1. function; 2. structure prediction
254780224	cytoplasmic	4	structure prediction
254781214	cytoplasmic	4	
254781048	cytoplasmic	3	function
254781039	cytoplasmic	3	function
254780227	cytoplasmic	3	function

254780137	cytoplasmic	3	1. function; 2. structure prediction
254780544	cytoplasmic	3	1. function; 2. structure prediction
254780794	cytoplasmic	3	1. function; 2. structure prediction
254780812	cytoplasmic	3	1. function; 2. structure prediction
254780821	cytoplasmic	3	1. function; 2. structure prediction
254780869	cytoplasmic	3	1. function; 2. structure prediction
254781017	cytoplasmic	3	1. function; 2. structure prediction
254781038	cytoplasmic	3	1. function; 2. structure prediction
peg_33	cytoplasmic	3	1. function; 2. structure prediction
254780845	cytoplasmic	3	1. function; 2. structure prediction
254780964	cytoplasmic	3	1. function; 2. structure prediction
peg_888	cytoplasmic	3	1. function; 2. structure prediction
254780662	cytoplasmic	3	pfam
254780446	cytoplasmic	3	
254780449	cytoplasmic	3	
254780546	cytoplasmic	3	structure prediction
254780994	cytoplasmic	3	
254781001	cytoplasmic	3	
254781024	cytoplasmic	3	structure prediction
254781083	cytoplasmic	3	
254780840	cytoplasmic	3	
254780861	cytoplasmic	3	structure prediction??
254780195	cytoplasmic	2	function
254780439	cytoplasmic	2	function
254780920	cytoplasmic	2	function
254781031	cytoplasmic	2	function
254780282	cytoplasmic	2	1. function; 2. structure prediction
254780420	cytoplasmic	2	1. function; 2. structure prediction
254780465	cytoplasmic	2	1. function; 2. structure prediction
254780488	cytoplasmic	2	1. function; 2. structure prediction
254780497	cytoplasmic	2	1. function; 2. structure prediction
254780503	cytoplasmic	2	1. function; 2. structure prediction
254780535	cytoplasmic	2	1. function; 2. structure prediction
254780655	cytoplasmic	2	1. function; 2. structure prediction
254780673	cytoplasmic	2	1. function; 2. structure prediction
254780718	cytoplasmic	2	1. function; 2. structure prediction
254780743	cytoplasmic	2	function
254781000	cytoplasmic	2	1. function; 2. structure prediction
254781004	cytoplasmic	2	1. function; 2. structure prediction
254780129	cytoplasmic	2	1. function; 2. structure prediction
peg_34	cytoplasmic	2	1. function; 2. structure prediction
254780156	cytoplasmic	2	1. function; 2. structure prediction
254780157	cytoplasmic	2	1. function; 2. structure prediction
254780186	cytoplasmic	2	1. function; 2. structure prediction
254780215	cytoplasmic	2	1. function; 2. structure prediction
254780258	cytoplasmic	2	1. function; 2. structure prediction
254780270	cytoplasmic	2	1. function; 2. structure prediction
254780272	cytoplasmic	2	1. function; 2. structure prediction
254780280	cytoplasmic	2	1. function; 2. structure prediction
254780283	cytoplasmic	2	1. function; 2. structure prediction
254780290	cytoplasmic	2	1. function; 2. structure prediction
255764462	cytoplasmic	2	1. function; 2. structure prediction
254780316	cytoplasmic	2	1. function; 2. structure prediction

254780325	cytoplasmic	2	1. function; 2. structure prediction
254780334	cytoplasmic	2	1. function; 2. structure prediction
254780346	cytoplasmic	2	1. function; 2. structure prediction
254780348	cytoplasmic	2	1. function; 2. structure prediction
254780355	cytoplasmic	2	1. function; 2. structure prediction
254780383	cytoplasmic	2	1. function; 2. structure prediction
254780392	cytoplasmic	2	1. function; 2. structure prediction
254780414	cytoplasmic	2	1. function; 2. structure prediction
254780458	cytoplasmic	2	1. function; 2. structure prediction
254780479	cytoplasmic	2	1. function; 2. structure prediction
254780486	cytoplasmic	2	1. function; 2. structure prediction
254780489	cytoplasmic	2	1. function; 2. structure prediction
254780820	cytoplasmic	2	1. function; 2. structure prediction
254780807	cytoplasmic	2	1. function; 2. structure prediction
254780791	cytoplasmic	2	1. function; 2. structure prediction
254780771	cytoplasmic	2	1. function; 2. structure prediction
254780767	cytoplasmic	2	1. function; 2. structure prediction
254780744	cytoplasmic	2	1. function; 2. structure prediction
254780684	cytoplasmic	2	1. function; 2. structure prediction
255764490	cytoplasmic	2	1. function; 2. structure prediction
254780653	cytoplasmic	2	1. function; 2. structure prediction
254780638	cytoplasmic	2	1. function; 2. structure prediction
255764494	cytoplasmic	2	1. function; 2. structure prediction
254780619	cytoplasmic	2	1. function; 2. structure prediction
255764495	cytoplasmic	2	1. function; 2. structure prediction
254780605	cytoplasmic	2	1. function; 2. structure prediction
254780604	cytoplasmic	2	1. function; 2. structure prediction
254780588	cytoplasmic	2	1. function; 2. structure prediction
254780521	cytoplasmic	2	function
254780498	cytoplasmic	2	1. function; 2. structure prediction
254780842	cytoplasmic	2	1. function; 2. structure prediction
254780849	cytoplasmic	2	1. function; 2. structure prediction
254780853	cytoplasmic	2	1. function; 2. structure prediction
254780868	cytoplasmic	2	1. function; 2. structure prediction
254780872	cytoplasmic	2	1. function; 2. structure prediction
254780873	cytoplasmic	2	1. function; 2. structure prediction
254780889	cytoplasmic	2	1. function; 2. structure prediction
254780900	cytoplasmic	2	1. function; 2. structure prediction
254780923	cytoplasmic	2	function
254780927	cytoplasmic	2	1. function; 2. structure prediction
254780942	cytoplasmic	2	1. function; 2. structure prediction
254780943	cytoplasmic	2	1. function; 2. structure prediction
254780956	cytoplasmic	2	1. function; 2. structure prediction
254780960	cytoplasmic	2	1. function; 2. structure prediction
254781019	cytoplasmic	2	1. function; 2. structure prediction
255764509	cytoplasmic	2	1. function; 2. structure prediction
254781026	cytoplasmic	2	1. function; 2. structure prediction
255764510	cytoplasmic	2	1. function; 2. structure prediction
254781047	cytoplasmic	2	1. function; 2. structure prediction
254781099	cytoplasmic	2	1. function; 2. structure prediction
254781101	cytoplasmic	2	1. function; 2. structure prediction
254781102	cytoplasmic	2	1. function; 2. structure prediction
254781176	cytoplasmic	2	1. function; 2. structure prediction

255764515	cytoplasmic	2	1. function; 2. structure prediction
254780858	cytoplasmic	2	structure prediction
254780883	cytoplasmic	2	
254780998	cytoplasmic	2	structure prediction
254781096	cytoplasmic	2	structure prediction
254780855	cytoplasmic	2	structure prediction
254780139	cytoplasmic	1	1. function; 2. structure prediction
254780242	cytoplasmic	1	1. function; 2. structure prediction
254780273	cytoplasmic	1	1. function; 2. structure prediction
254780328	cytoplasmic	1	1. function; 2. structure prediction
254780337	cytoplasmic	1	1. function; 2. structure prediction
254780404	cytoplasmic	1	1. function; 2. structure prediction
254780526	cytoplasmic	1	1. function; 2. structure prediction
254780538	cytoplasmic	1	1. function; 2. structure prediction
254780921	cytoplasmic	1	1. function; 2. structure prediction
254781062	cytoplasmic	1	1. function; 2. structure prediction
254781091	cytoplasmic	1	1. function; 2. structure prediction
255764513	cytoplasmic	1	1. function; 2. structure prediction
254780413	cytoplasmic	1	structure prediction
254780915	cytoplasmic	1	structure prediction
254780130	cytoplasmic	1	
254780143	cytoplasmic	1	
254780144	cytoplasmic	1	
254780145	cytoplasmic	1	
254780147	cytoplasmic	1	
254780151	cytoplasmic	1	
254780153	cytoplasmic	1	
254780158	cytoplasmic	1	
254780163	cytoplasmic	1	
254780166	cytoplasmic	1	
254780168	cytoplasmic	1	
254780180	cytoplasmic	1	
254780182	cytoplasmic	1	
254780184	cytoplasmic	1	
254780191	cytoplasmic	1	
254780201	cytoplasmic	1	
254780217	cytoplasmic	1	
254780233	cytoplasmic	1	
254780234	cytoplasmic	1	
254780238	cytoplasmic	1	
254780259	cytoplasmic	1	
254780261	cytoplasmic	1	
254780264	cytoplasmic	1	
254780267	cytoplasmic	1	
254780269	cytoplasmic	1	
254780271	cytoplasmic	1	
254780274	cytoplasmic	1	
254780279	cytoplasmic	1	
254780293	cytoplasmic	1	
254780297	cytoplasmic	1	
254780299	cytoplasmic	1	
254780301	cytoplasmic	1	
254780309	cytoplasmic	1	

254780310	cytoplasmic	1	
254780322	cytoplasmic	1	
254780326	cytoplasmic	1	
254780327	cytoplasmic	1	
254780329	cytoplasmic	1	
254780336	cytoplasmic	1	
254780339	cytoplasmic	1	
254780345	cytoplasmic	1	
254780353	cytoplasmic	1	
254780363	cytoplasmic	1	
254780364	cytoplasmic	1	
254780365	cytoplasmic	1	
254780370	cytoplasmic	1	
254780406	cytoplasmic	1	
254780409	cytoplasmic	1	
254780410	cytoplasmic	1	
254780418	cytoplasmic	1	
254780430	cytoplasmic	1	
254780432	cytoplasmic	1	
254780434	cytoplasmic	1	
254780457	cytoplasmic	1	
254780460	cytoplasmic	1	
254780462	cytoplasmic	1	
254780478	cytoplasmic	1	
254780480	cytoplasmic	1	
peg_394	cytoplasmic	1	
254780828	cytoplasmic	1	
254780826	cytoplasmic	1	
254780823	cytoplasmic	1	
peg_409	cytoplasmic	1	
254780809	cytoplasmic	1	
254780806	cytoplasmic	1	
254780804	cytoplasmic	1	
254780802	cytoplasmic	1	
255764478	cytoplasmic	1	
254780784	cytoplasmic	1	
254780778	cytoplasmic	1	
254780777	cytoplasmic	1	
254780775	cytoplasmic	1	
255764481	cytoplasmic	1	
254780768	cytoplasmic	1	
254780755	cytoplasmic	1	
254780754	cytoplasmic	1	
254780750	cytoplasmic	1	
254780742	cytoplasmic	1	
254780725	cytoplasmic	1	
254780716	cytoplasmic	1	
254780715	cytoplasmic	1	
254780710	cytoplasmic	1	
254780709	cytoplasmic	1	
254780689	cytoplasmic	1	
254780682	cytoplasmic	1	
254780676	cytoplasmic	1	

254780675	cytoplasmic	1	
254780674	cytoplasmic	1	
254780669	cytoplasmic	1	
254780644	cytoplasmic	1	
254780630	cytoplasmic	1	
254780629	cytoplasmic	1	
254780625	cytoplasmic	1	
254780622	cytoplasmic	1	
254780618	cytoplasmic	1	
254780616	cytoplasmic	1	
254780613	cytoplasmic	1	
254780610	cytoplasmic	1	
254780601	cytoplasmic	1	
255764496	cytoplasmic	1	
254780596	cytoplasmic	1	
254780591	cytoplasmic	1	
254780582	cytoplasmic	1	
254780559	cytoplasmic	1	
254780554	cytoplasmic	1	
254780553	cytoplasmic	1	
254780536	cytoplasmic	1	
254780533	cytoplasmic	1	
254780506	cytoplasmic	1	
254780505	cytoplasmic	1	
254780502	cytoplasmic	1	
peg_753	cytoplasmic	1	
255764505	cytoplasmic	1	
254780871	cytoplasmic	1	
peg_817	cytoplasmic	1	
254780902	cytoplasmic	1	
254780904	cytoplasmic	1	
254780905	cytoplasmic	1	
254780918	cytoplasmic	1	
254780936	cytoplasmic	1	
254780940	cytoplasmic	1	
254780945	cytoplasmic	1	
254780947	cytoplasmic	1	
254780948	cytoplasmic	1	
254780949	cytoplasmic	1	
peg_887	cytoplasmic	1	
254780970	cytoplasmic	1	
254780971	cytoplasmic	1	
254780976	cytoplasmic	1	
254780978	cytoplasmic	1	
254780991	cytoplasmic	1	
254781012	cytoplasmic	1	
254781016	cytoplasmic	1	
254781020	cytoplasmic	1	
254781028	cytoplasmic	1	
254781040	cytoplasmic	1	
254781049	cytoplasmic	1	
254781050	cytoplasmic	1	
254781052	cytoplasmic	1	

254781054	cytoplasmic	1	
254781055	cytoplasmic	1	
254781060	cytoplasmic	1	
254781071	cytoplasmic	1	
254781076	cytoplasmic	1	
254781077	cytoplasmic	1	
254781089	cytoplasmic	1	
254781093	cytoplasmic	1	
254781095	cytoplasmic	1	
254781106	cytoplasmic	1	
254781107	cytoplasmic	1	
254781111	cytoplasmic	1	
254781149	cytoplasmic	1	
peg_1087	cytoplasmic	1	
254781177	cytoplasmic	1	
254781187	cytoplasmic	1	
254781202	cytoplasmic	1	
254781206	cytoplasmic	1	
254781208	cytoplasmic	1	
254781215	cytoplasmic	1	

N: number of methods (out of 6) that predict the protein to have transmembrane helix (helices)
SP: signal peptide
TMH: transmembrane helix

Cell color:
Light blue: transmembrane proteins that are located in the inner membrane of this Gram-negative bacterium.
Light green: proteins with signal peptides and they are likely function in the extracytoplasmic space;
Yellow: extracytoplasmic proteins that are without signal peptides and that are secreted in Sec-independent pathways;
Pink: cytoplasmic proteins
