# Supplementary material for: Predictive Sequence Analysis of the Candidatus Liberibacter asiaticus Proteome
Source: PLoS One. 2012 Jul 18;7(7):e41071. doi: 10.1371/journal.pone.0041071 (PMC3399792; doi:10.1371/journal.pone.0041071)
Supplement: Table S4 — Percentage of duplicated genes in proteomes of similar size to Ca. L. asiaticus. (PDF) [file pone.0041071.s004.pdf]

Table S4. Percentage of duplicated genes in the proteomes with similar size to *C. L. asiaticus*

species name	proteome size	percentage of duplicated genes
**Orientia tsutsugamushi str. Boryong**	1182	50.931
**Mycoplasma mycoides subsp. mycoides SC str.**	1095	48.676
**Mycoplasma mycoides subsp. mycoides SC str. PG1**	1016	45.768
**Lactobacillus fermentum CECT 5716**	1051	45.576
**Burkholderia vietnamiensis G4**	1114	44.075
**Mycoplasma penetrans HF-2**	1037	40.405
**Wolbachia sp. wRi**	1150	39.391
**Brucella microti CCM 4915**	1167	36.675
**Wolbachia endosymbiont of Drosophila**	1195	34.895
**Vibrio cholerae O395**	1133	32.304
**Candidatus Liberibacter solanacearum CLso-ZC1**	1192	31.208
**Bartonella quintana str. Toulouse**	1142	26.620
**cyanobacterium UCYN-A**	1199	26.439
**Chlamydophila pneumoniae J138**	1069	26.099
**Lawsonia intracellularis PHE/MN1-00**	1185	25.907
**Chlamydophila pneumoniae CWL029**	1052	25.665
**Chlamydophila pneumoniae TW-183**	1113	24.618
**Chlamydophila pneumoniae LPCoLN**	1097	24.521
**Chlamydophila pneumoniae AR39**	1110	24.234
**Chlamydophila felis Fe/C-56**	1005	23.184
**Ehrlichia chaffeensis str. Arkansas**	1105	23.167
**Treponema pallidum subsp. pallidum str. Nichols**	1031	22.599
**Candidatus Liberibacter asiaticus str. psy62**	1109	22.092
**Treponema pallidum subsp. pallidum SS14**	1028	22.082
**Rickettsia africae ESF-5**	1030	21.553
**Rickettsia canadensis str. McKiel**	1093	16.926
