# Supplementary material for: Predictive Sequence Analysis of the Candidatus Liberibacter asiaticus Proteome
Source: PLoS One. 2012 Jul 18;7(7):e41071. doi: 10.1371/journal.pone.0041071 (PMC3399792; doi:10.1371/journal.pone.0041071)
Supplement: Table S5 — Homologous groups in Ca. L.asiaticus proteome. (PDF) [file pone.0041071.s005.pdf]

Table S5. Homologous groups in *Ca. L .asiaticus* proteome

Homologous group1: ABC-type ATPase		
254780139	ABC transporter ATPase protein	
254780173	ABC transporter ATPase protein	
254780193	ABC transporter ATPase protein and transmembrane protein	
254780273	ABC transporter ATPase homolog	Likely function as transcriptional regulator
254780340	ABC transporter ATPase protein	
255764467	ABC transporter ATPase protein and transmembrane protein	Function in type I secretion
254780538	ABC transporter ATPase protein	
254780559	ABC transporter ATPase protein	
254780576	ABC transporter ATPase protein and transmembrane protein	
254780596	ABC transporter ATPase protein	
254780704	ABC transporter ATPase protein	
254780718	ABC transporter ATPase protein	
254780744	ABC transporter ATPase protein	
254780871	ABC transporter ATPase protein	
254780917	ABC transporter ATPase protein and transmembrane protein	
254781060	SufC, ABC transporter ATPase homolog	Involve in Fe-S cluster assembly
254781123	ABC transporter ATPase homolog	Likely function as transcriptional regulator
Homologous group 2: GTP-binding protein		
254780150	translation elongation factor Tu	
254780233	GTP-binding protein	
254780263	translation elongation factor Tu	
254780264	elongation factor G	
254780321	GTP-binding protein LepA	
254780787	translation initiation factor IF-2	
Homologous group 3: pilin component		
254780736	Flp/Fap pilin component	
254780735	Flp/Fap pilin component	
254780734	Flp/Fap pilin component	
254780733	Flp/Fap pilin component	
254780732	Flp/Fap pilin component	
254780730	Flp/Fap pilin component	
Homologous group 4: two component sensor, histidine kinase		
254780312	two-component sensor histidine kinase protein	
254780413	two-component sensor histidine kinase protein	
254780450	two-component sensor histidine kinase protein	
255764476	two-component sensor histidine kinase protein	
254780701	two-component sensor histidine kinase protein	
254780903	two-component sensor histidine kinase protein	
Homologous group 5: helicase		
254780382	ATP dependent RNA helicase protein	
254780619	primosome assembly protein PriA, has 3'-5' helicase activity	
254780601	ATP-dependent RNA helicase protein	
254780945	ATP-dependent DNA helicase RecG	
254780947	transcription-repair coupling factor, superfamily II helicase	
Homologous group 6: Amino-acid-tRNA sythetase		
254780445	isoleucyl-tRNA synthetase	
254780802	leucyl-tRNA synthetase	
254780933	valyl-tRNA synthetase	
255764461	methionyl-tRNA synthetase	
Homologous group 7: oxidoreductase		
254780462	enoyl-(acyl carrier protein) reductase	
254780535	3-ketoacyl-(acyl-carrier-protein) reductase	
254781166	enoyl-(acyl carrier protein) reductase	
254780337	oxidoreductase protein	
Homologous group 8: ATP synthase proteins		

254780615	F0F1 ATP synthase subunit beta	
254780617	F0F1 ATP synthase subunit alpha	
254780684	flagellum-specific ATP synthase	
254780810	transcription termination factor Rho	Homolog, but not the same function
Homologous group 9: ATP-dependent protease component		
254780163	ATP-dependent Clp protease ATP-binding subunit ClpA	
254780271	ATP-dependent protease ATP-binding subunit ClpX	
254780829	ATP-dependent protease ClpYQ, ATPase subunit	
254780877	ATP-dependent Clp protease ATP-binding subunit ClpA	
Homologous group 10: NADH dehydrogenase subunits		
254780865	NADH dehydrogenase subunit L	
254780866	NADH dehydrogenase subunit M	
254780867	NADH dehydrogenase subunit N	
Homologous group 11: ABC transporter membrane spanning protein		
254780171	ABC transporter membrane spanning protein	
254780172	ABC transporter membrane spanning protein	
254781113	ABC transporter membrane spanning protein	
Homologous group 12: amino-acid-tRNA synthetase		
254781118	lysyl-tRNA synthetase	
254781066	Truncated, possibly inactive lysyl-tRNA synthetase protein	
254780419	aspartyl-tRNA synthetase	
Homologous group 13: ABC transporter membrane spanning protein		
254780539	ABC transporter, membrane spanning protein	
254780540	ABC transporter, membrane spanning protein	
254780719	zinc uptake ABC transporter, permease protein	
Homologous group 14: GTP-binding protein		
255764471	GTP-binding protein EngA	
254780941	GTP-binding protein ErA	
254780809	tRNA modification GTPase TrmE	
Homologous group 15: Pyruvate/2-oxoglutarate dehydrogenase complex component		
254780673	pyruvate dehydrogenase complex subunit	
254780674	pyruvate dehydrogenase complex subunit	
254781052	dihydrolipoamide succinyltransferase	
Homologous group 16: Pyruvate/2-oxoglutarate dehydrogenase complex component		
254781053	dihydrolipoamide dehydrogenase	
254781055	glutathione reductase	
254780675	dihydrolipoamide dehydrogenase	
Homologous group 17: Lyase		
254780190	fumarate hydratase	
254780370	argininosuccinate lyase	
254780974	adenylosuccinate lyase	
Homologous group 18: flagellar components		
254780378	flagellar basal body rod protein FlgG	
254780525	flagellar hook protein FlgE	
254780685	flagellar basal body rod protein FlgF	
Homologous group 19: hydroxylase		
254780168	2-polyprenyl-6-methoxyphenyl hydroxylase	
254780842	2-octaprenyl-6-methoxyphenyl hydroxylase	
Homologous group 20: DNA topoisomerase IV subunit A		
254780182	DNA topoisomerase IV subunit A	
254780353	DNA topoisomerase IV subunit A	
Homologous group 21: glutamine syntetase		
254780185	glutamine synthetase	
254780503	glutamine synthetase	
Homologous group 22: lipopolysaccharide biosynthesis protein		
254780201	lipopolysaccharide biosynthesis protein	
254780923	lipopolysaccharide biosynthesis protein	
Homologous group 23: aminotransferase		

254780486	8-amino-7-oxononanoate synthase	
254780604	5-aminolevulinate synthase	
Homologous group 24: enzymes in fatty acid synthesis		
254780489	3-oxoacyl-(acyl carrier protein) synthase II, FabH	
254780497	3-oxoacyl-(acyl carrier protein) synthase III, FabH	
Homologous group 25: transcriptional regulator		
254780526	putative transcription regulator protein	
254780893	transcriptional regulator	
Homologous group 26: formyltransferase		
254780570	phosphoribosylglycinamide formyltransferase	
254780911	formyltetrahydrofolate deformylase	
Homologous group 27: hydrolase		
254780583	hydroxyacylglutathione hydrolase	
254780758	Zn-dependent hydrolase	
Homologous group 28: cell division protein		
254780606	cell division protein	
254780799	DNA translocase FtsK	
Homologous group 29: RNA-pseudouridylate synthase protein		
254780608	RNA-pseudouridylate synthase protein, ribosomal large subunit C	
254780610	RNA-pseudouridylate synthase protein, ribosomal large subunit D	
Homologous group 30: tRNA and rRNA methylase		
254780634	tRNA and rRNA methylase	
254780666	tRNA and rRNA methylase	
Homologous group 31: transcriptional regulator		
254780693	transcriptional regulator	
254780694	transcriptional regulator	
Homologous group 32: ABC transporter transmembrane protein		
254780705	ABC transporter transmembrane protein	
255764486	ABC transporter transmembrane protein	
Homologous group 33: signal recognition particle protein		
254780709	signal recognition particle-docking protein	Involve in cell division
254780711	signal recognition particle protein	
Homologous group 34: peptide chain release factor		
254780753	peptide chain release factor 2	
254780874	peptide chain release factor 1	
Homologous group 35: Polyprenyl synthetase		
254780755	octaprenyl-diphosphate synthase protein	
254781111	geranyltranstransferase protein	
Homologous group 36: acyltransferase		
254780771	UDP-3-O-[3-hydroxymyristoyl] glucosamine N-acyltransferase	
255764481	UDP-N-acetylglucosamine acyltransferase	
Homologous group 37: tRNA synthetase		
254780870	prolyl-tRNA synthetase	
254781004	threonyl-tRNA synthetase	
Homologous group 38: dehydrogenase E1 subunit		
254781051	2-oxoglutarate dehydrogenase, E1 subunit	
255764490	pyruvate dehydrogenase, E1 subunit	
Homologous group 39: iron-sulfur cluster assembly protein		
254781059	iron-sulfur cluster assembly protein, sufB	
254781061	iron-sulfur cluster assembly protein, sufD	
Homologous group 40: desulfurase		
254781062	selenocysteine lyse/cysteine desulfurase	
254781091	cystein desulfurase	
Homologous group 41: enzymes in Peptidoglycan Biosynthesis		
254781101	UDP-N-acetylmuramoylalanyl-D-glutamyl-2, 6-diaminopimelate--D-alanyl-D-alanyl ligase, murF	
254781102	UDP-N-acetylmuramoylalanyl-D-glutamate--2,6-diaminopimelate	

	ligase, murE	
Homologous group 42: ribonuclease		
254780630	ribonuclease PH	share one domain with 254780784
254780784	polynucleotide phosphorylase/polyadenylase	share one domain with 254780630
Homologous group 43: ABC-transporter substrate binding protein		
254780537	ABC-transporter substrate-binding protein	
254780717	ABC-transporter substrate-binding protein	
Homologous group 44: mutase		
254780544	phosphoglucosamine mutase	
254781107	phosphoglucomutase	
Homologous group 45: DNA topoisomerase IV subunit B		
254780222	DNA topoisomerase IV subunit B	
254780814	DNA topoisomerase IV subunit B	
Homologous group 46: GTPase		
254780226	translation-associated GTPase	
254780648	GTPase ObgE involve in DNA replication	
Homologous group 47: proteins with ATP grasp domain.		
254780268	acetyl-CoA carboxylase biotin carboxylase subunit	
254780439	carbamoyl phosphate synthase large subunit	
Homologous group 48: iron-sulfur cluster assembly protein		
254780287	iron-sulfur cluster assembly accessory protein	
254781064	FeS assembly scaffold SufA	
Homologous group 49: RNA polymerase sigma factor		
254780289	RNA polymerase sigma factor RpoD	
254780611	RNA polymerase factor sigma-32	
Homologous group 50: carbamoyltransferase		
254780300	aspartate carbamoyltransferase catalytic subunit	
255764473	ornithine carbamoyltransferase	
Homologous group 51: NAD-dependent epimerase/dehydratase		
254780328	UDP-glucose 4-epimerase	
254780920	dTDP-glucose 4,6-dehydratase	
Homologous group 52: replicative DNA helicase		
254780332	replicative DNA helicase	
254781149	putative replicative DNA helicase	shorter than homologour proteins
Homologous group 53: amido/amino-transferase		
254780336	amidophosphoribosyltransferase	
254780943	glucosamine--fructose-6-phosphate aminotransferase	
Homologous group 54: glucose-1-phosphate NTP_transferase		
254780365	UTP-glucose-1-phosphate uridylyltransferase protein	
254780922	glucose-1-phosphate thymidylyltransferase	
Homologous group 55: base excision repair protein		
254780383	endonuclease III	
254780479	A/G-specific adenine glycosylase	
Homologous group 56: transporter		
254780387	C4-dicarboxylate transporter	
254781171	transporter	
Homologous group 57: fatty acid synthesis protein		
254780461	3-oxoacyl-(acyl carrier protein) synthase I, FabB	
254780533	3-oxoacyl-(acyl carrier protein) synthase II, FabF	
Homologous group 58: transaminase		
254780432	acetylornithine transaminase protein	
254780488	adenosylmethionine--8-amino-7-oxononanoate transaminase	
Homologous group 59: D-alanyl-D-alanine carboxypeptidase		
254780435	D-alanyl-D-alanine carboxypeptidase 1	
254780698	D-alanyl-D-alanine carboxypeptidase 1 penicillin-binding protein	
Homologous group 60: unknown		
254780448	unknown	

254780449	unknown	
Homologous group 61: GGDEF domain containing protein		
254780468	sensory box/GGDEF family protein	
254781079	GGDEF domain containing protein or diguanylate cyclase	
Homologous group 62: ferredoxin-NADP+ reductase		
254780363	ferredoxin-NADP+ reductase protein	
254780364	ferredoxin-NADP+ reductase protein	
Homologous group 63: unknown		
254780122	unknown	likely to be from the integrated SC1 prophage
254781220	unknown	likely to be from the integrated SC1 prophage
Homologous group 64: uknown		
254780124	unknown	likely to be from the integrated SC1 prophage
254781191	unknown	
Homologous group 65:prophage antirepressor		
254780125	prophage antirepressor	likely to be from the integrated SC1 prophage
254780555	unknown	aligned to piece of 254780125
254780987	prophage antirepressor	
Homologous group 66: DNA-binding protein		
254780126	phage protein, may related to DNA binding	likely to be from the integrated SC1 prophage
254781192	single-strand DNA binding protein	
Homologous group 67: prophage DNA polymerase		
254780127	prophage DNA polymerase	likely to be from the integrated SC1 prophage
254781193	prophage DNA polymerase	
Homologous group 68: restriction endonuclease		
254780128	restriction endonuclease	likely to be from the integrated SC1 prophage
254781194	restriction endonuclease	
Homologous group 69: SNF2 family DNA/RNA helicase		
254780129 + 254780130	SNF2 family DNA/RNA helicases	(1)They are the boundary of the integrated prophage and 254780129 is likely to belong to the prophage. (2)Two neighbouring proteins both aligned to part of 255764515. They may function indenpendently or cooperatively. It is also possible they are psuedogenes.
255764515	DNA/RNA helicases, SNF2 family	It is the boundary of the integrated prophage, the C-terminus half of the protein is likely to be belong to the prophage
Homologous group 70: NAD-dependant DNA ligase		
254780131	NAD-dependant DNA ligase	aligned to one domain of 254781172, 99% identical to 254781196, near the region of integrated prophage
254781172	NAD-dependant DNA ligase	
254781196	NAD-dependant DNA ligase	likely to be from the brophage, 99% identical to 254780131, aligned to one domain of 254781172
Homologous group 71: Guanylate kinase		
254780132	guanylate kinase	near the region of the integrated SC1 prophage
254781197	guanylate kinase	likely from the prophage, 98% identical to 254780132
255764507	guanylate kinase	
Homologous group 72: unknown		
254780988	unknown	
254781199	unknown	likely to be from the SC1 prophage
Homologous group 73: phage terminase, large subunit		
254781187	phage terminase, large subunit	
254781215	phage terminase, large subunit	likely to be from the SC1 prophage
Homologous group 74: unknown		
254781188	unknown	
254781217	unknown	likely to be from the SC1 prophage
Homologous group 75: primase		
254781190	primase-like protein	only aligned to part of 254781125
254781225	P4 family phage/plasmid primase	likely to be from the SC1 prophage
Homologous group 76: ribonucleotide-diphosphate reductase subunit beta		
254780136	ribonucleotide-diphosphate reductase subunit beta	100% identical to a piece of 254780845 or 254780964, close to the region of mapped prophage

254780845	ribonucleotide-diphosphate reductase subunit beta	
254780964	ribonucleotide-diphosphate reductase subunit beta	
254781006	ribonucleotide-diphosphate reductase subunit beta	100% identical to a piece of 254780845 or 254780964
254781153	ribonucleotide-diphosphate reductase subunit beta	just a small piece of 254780845 or 254780964, 95% identity
Homologous group 77: unknown protein from the prophage		
255764516	unknown	likely to be from the SC1 prophage
255764517	unknown	likely to be from the SC1 prophage
Homologous group 78: GCN5-related N-acetyltransferase		
254780989	GCN5-related N-acetyltransferase	
254780962	GCN5-related N-acetyltransferase	64% identical to piece of 254780989
254781162	GCN5-related N-acetyltransferase	
254781155	GCN5-related N-acetyltransferase	64% identical to piece of 254780989
Homologous group 79: phage-related lysozyme		
254781057 + 254781058	phage-related lysozyme	neighbouring proteins both aligned to part of 254781056. They may function indenpendently or cooperatively. It is also possible they are psuedogenes
254781056	phage-related lysozyme	
Homologous group 80: unknown proteins		
254780492	Unknown	40 aa short gene, 100% identical to 254780880 and 254780830
254780830	Unknown	40 aa short gene, 100% identical to 254780492 and 254780880
254780880	Unknown	40 aa short gene, 100% identical to 254780492 and 254780830
Homologous group 81: ATPase involved in chromosome partitioning		
254780491	ATPase involved in chromosome partitioning	100% identical to the C-terminal domain of 254780831
254780831	ATPase involved in chromosome partitioning	
254780881	ATPase involved in chromosome partitioning	100% identical to the C-terminal domain of 254780831
Homologous group 82: site-specific tyrosine recombinase		
254780490	site-specific tyrosine recombinase XerC	90% identical to the N-terminal domain of 254780882
255764498	site-specific tyrosine recombinase XerD	
254780882	site-specific tyrosine recombinase XerC	
Homologous group 83: von Willebrand factor type A domain containing protein		
254780388	von Willebrand factor type A	
254780833	von Willebrand factor type A	
254780934	von Willebrand factor type A	
254781108	von Willebrand factor type A	
254781110	von Willebrand factor type A	
Homologous group 84: unknown proteins		
254780203 + 254780204	unknown	Two neighbouring proteins aligned to different regions of 254781189. They may function indenpendently or cooperatively. It is also possible that they are psuedogenes
254781124 + 254781125	unknown	Two neighbouring proteins aligned to different regions of 254781189. They may function indenpendently or cooperatively. It is also possible that they are psuedogenes
254781189	unknown	Transmembrane protein, fast evolution
Homologous group 85: unknown proteins		
254780135	Unknown	has signal peptide, fast-evolving
254781007	Unknown	has signal peptide, fast-evolving
254780914	Unknown	has signal peptide, fast-evolving
254780929	Unknown	has signal peptide, fast-evolving
Homologous group 86: Endonuclease/exonuclease/phosphatase family proteins		
254780797	Endonuclease/exonuclease/phosphatase family	
254780995	Endonuclease/exonuclease/phosphatase family	
254780996	Endonuclease/exonuclease/phosphatase family	
254781003	Endonuclease/exonuclease/phosphatase family	has signal peptide
Homologous group 87: unknown proteins		
254780984	Unknown	has signal peptide, fast evolving protein
254781126	Unknown	
254780981	Unknown	has signal peptide, fast evolving protein
Homologous group 88: CBS domain containing protein		

254780426	CBS-domain containing protein, hemolysin-like	Might be exported by Type I secretion system and function as virulence factor
255764470	CBS-domain containing transmembrane protein	
Homologous group 89:unknown		
254780556	unknown	has signal peptide, fast evolving protein
254780980	unknown	has signal peptide, fast evolving protein
Homologous group 90:		
254780886	unknown	has signal peptide, fast evolving protein
254781005	unknown	has signal peptide, fast evolving protein
