# Supplementary material for: Predictive Sequence Analysis of the Candidatus Liberibacter asiaticus Proteome
Source: PLoS One. 2012 Jul 18;7(7):e41071. doi: 10.1371/journal.pone.0041071 (PMC3399792; doi:10.1371/journal.pone.0041071)
Supplement: Table S6 — Ca. L. asiaticus Proteins with abnormal evolutionary history. (PDF) [file pone.0041071.s006.pdf]

Table S6: Proteins with abnormal evolutionary history

| Proteins from the integrated SC1 prophage | | | | | |
|---|---|---|---|---|---|
| **NCBI gi** | **Organism of the closest homolog** | **Class** | **Kingdom** | **Function prediction** | **Comments** |
| 255764515 | Liberibacter SC1 phage | | Viruses | DNA/RNA helicase, SNF2 family | C-terminal half belongs to the phage |
| 254781196 | Liberibacter SC1 phage | | Viruses | NAD-dependent DNA ligase | |
| 254781197 | Liberibacter SC1 phage | | Viruses | guanylate kinase | Duplication upon phage integration |
| 254781198 | Liberibacter SC1 phage | | Viruses | unknown | |
| 254781199 | Liberibacter SC1 phage | | Viruses | transmembrane protein | |
| 255764516 | Liberibacter SC1 phage | | Viruses | unknown | |
| 255764517 | Liberibacter SC1 phage | | Viruses | unknown | |
| 254781202 | Liberibacter SC1 phage | | Viruses | Structure protein of bacteriophage | Virus protein |
| 254781203 | Liberibacter SC1 phage | | Viruses | unknown | |
| 254781204 | Liberibacter SC1 phage | | Viruses | minor structure protein | Virus protein |
| 254781205 | Liberibacter SC1 phage | | Viruses | unknown | |
| 254781206 | Liberibacter SC1 phage | | Viruses | unknown | |
| 254781207 | Liberibacter SC1 phage | | Viruses | unknown | |
| 254781208 | Liberibacter SC1 phage | | Viruses | unknown | |
| 254781209 | Liberibacter SC1 phage | | Viruses | phage related protein | Virus protein |
| 254781210 | Liberibacter SC1 phage | | Viruses | Major capsid protein | Virus protein |
| 254781211 | Liberibacter SC1 phage | | Viruses | unknown | |
| 254781212 | Liberibacter SC1 phage | | Viruses | unknown | |
| 254781213 | Liberibacter SC1 phage | | Viruses | head-to-tail joining protein, phage | Virus protein |
| 254781214 | Liberibacter SC1 phage | | Viruses | unknown | |
| 254781215 | Liberibacter SC1 phage | | Viruses | phage terminase, large subunit | Virus protein |
| 254781216 | Liberibacter SC1 phage | | Viruses | unknown | |
| 254781217 | Liberibacter SC1 phage | | Viruses | Phage protein related to DNA packing | Virus protein |
| 254781218 | Liberibacter SC1 phage | | Viruses | transcriptional repressor | Virus protein |
| 254781219 | Liberibacter SC1 phage | | Viruses | unknown | |
| 254781220 | Liberibacter SC1 phage | | Viruses | unknown | |
| 254781221 | Liberibacter SC1 phage | | Viruses | unknown | |
| 254781222 | Liberibacter SC1 phage | | Viruses | acyltransferase, phage related | Virus protein |
| 254781223 | Liberibacter SC1 phage | | Viruses | phage associated protein | Virus protein |
| 254781224 | Liberibacter SC1 phage | | Viruses | transmembrane protein | |
| 254781225 | Liberibacter SC1 phage | | Viruses | P4 family phage/plasmid primase | Virus protein |
| 254781226 | Liberibacter SC1 phage | | Viruses | unknown | From Viruses |
| 254781227 | Liberibacter SC1 phage | | Viruses | unknown | |
| 254781228 | Liberibacter SC1 phage | | Viruses | unknown | |
| 254780122 | Liberibacter SC1 phage | | Viruses | unknown | |
| 254780123 | Liberibacter SC1 phage | | Viruses | unknown | |
| 254780124 | Liberibacter SC1 phage | | Viruses | phage-related, probably endo/exonuclease | Virus protein |
| 254780125 | Liberibacter SC1 phage | | Viruses | phage antirepressoer | Virus protein |
| 254780126 | Liberibacter SC1 phage | | Viruses | single-stranded DNA-binding protein | |
| 254780127 | Liberibacter SC1 phage | | Viruses | DNA polymerase from bacteriophage | Virus protein |
| 254780128 | Liberibacter SC1 phage | | Viruses | restriction endonuclease | |

| | | | | | |
|---|---|---|---|---|---|
| 254780129 | Liberibacter SC1 phage | | Viruses | DNA/RNA helicase, SNF2 family, N-terminal | |

Liberibacter proteins with duplicated version in the integrated *liberibacter SC1 phage*

| NCBI gi | Organism of the closest homolog | Class | Kingdom | Function prediction | Comments |
|---|---|---|---|---|---|
| 254780130 | Liberibacter SC1 phage | | Viruses | DNA/RNA helicase, SNF2 family, C-terminal | Duplicated upon phage integration |
| 254780132 | Liberibacter SC1 phage | | Viruses | guanylate kinase | Duplicated upon phage integration |

Potential phage proteins

| NCBI gi | Organism of the closest homolog | Class | Kingdom | Function prediction | Comments |
|---|---|---|---|---|---|
| 254780988 | Liberibacter SC1 phage | | Viruses | Unknown transmembrane protein | from another bacteriophage? |
| 254781013 | Salinibacter ruber | Sphingobacteria | Bacteria | phage related protein | from another bacteriophage? |
| 254781056 | Janthinobacterium sp. Marseille | Betaproteobacteria | Bacteria | phage-related lysozyme | from another bacteriophage? |
| 254781057 | Acidobacterium sp. MP5ACTX9 | Acidobacteria | Bacteria | phage-related protein | from another bacteriophage? |
| 254781058 | Janthinobacterium sp. Marseille | Betaproteobacteria | Bacteria | phage-related lysozyme | from another bacteriophage? |
| 254781187 | Liberibacter SC1 phage | | Viruses | phage terminase, large subunit | from another bacteriophage? |
| 254781186 | Liberibacter SC2 phage | | Viruses | Phage terminase, large subunit | Just piece of the protein |
| 254781188 | Liberibacter SC1 phage | | Viruses | phage protein, terminase small subunit | from another bacteriophage? |
| 254781190 | Liberibacter SC1 phage | | Viruses | primase | from another bacteriophage? |
| 254781191 | Liberibacter SC1 phage | | Viruses | unknown | from another bacteriophage? |
| 254781192 | Xylella fastidiosa | Gammaproteobacteria | Bacteria | phage-related protein | from another bacteriophage? |
| 254781193 | Escherichia coli | Gammaproteobacteria | Bacteria | DNA polymerase from bacteriophage | from another bacteriophage? |
| 254781194 | Liberibacter SC1 phage | | Viruses | Phage related endonuclease | from another bacteriophage? |

Proteins originated from bacteria out of the class *Alphaproteobacteria*

| NCBI gi | Organism of the closest homolog | Class | Kingdom | Function prediction | Comments |
|---|---|---|---|---|---|
| 254780144 | Anaeromyxobacter sp. Fw109-5 | Deltaproteobacteria | Bacteria | 50S ribosomal protein L12P | The top one is exception Most best hits are from Alphaproteobacteria |
| 254780229 | Thermobifida fusca | Actinobacteria (class) | Bacteria | helicase | |
| 254780386 | Halomonas elongate | Gammaproteobacteria | Bacteria | Type I secretion membrane protein | |
| 254780387 | Providencia stuartii | Gammaproteobacteria | Bacteria | C4-dicarboxylate transporter DctA | |
| 254780446 | Xylella fastidiosa | Gammaproteobacteria | Bacteria | unknown | |
| 254780735 | Arthrobacter chlorophenolicus | Actinobacteria (class) | Bacteria | pilin component | |
| 254780720 | Neptuniibacter caesariensis | Gammaproteobacteria | Bacteria | Ferroxidase | |
| 254780661 | Sodalis glossinidius | Gammaproteobacteria | Bacteria | exonuclease I | |
| 254780563 | Micromonospora sp. ATCC 39149 | Actinobacteria (class) | Bacteria | ABC-transporter extracellular solute-binding protein | fragment, too short to have sensible blast top hits |
| 254780562 | Xanthomonas oryzae | Gammaproteobacteria | Bacteria | unknown | |
| 254780561 | Photorhabdus luminescens | Gammaproteobacteria | Bacteria | thiamine transporter substrate binding subunit | |
| 255764501 | Arsenophonus nasoniae | Gammaproteobacteria | Bacteria | thiamine transporter membrane protein | |
| 254780559 | Arsenophonus nasoniae | Gammaproteobacteria | Bacteria | thiamine transporter ATP-binding subunit | |
| 255764504 | Neisseria lactamica | Betaproteobacteria | Bacteria | type II modification methyltransferase | |
| 254780516 | Neisseria lactamica | Betaproteobacteria | Bacteria | type II modification methyltransferase | |
| 254780515 | Neisseria lactamica | Betaproteobacteria | Bacteria | restriction enzyme | |
| 254780514 | Flavobacterium psychrophilum | Flavobacteria | Bacteria | type II restriction endonuclease | |
| 254780836 | Klebsiella pneumonia | Gammaproteobacteria | Bacteria | type I modification DNA methylase | |
| 254780837 | Klebsiella pneumonia | Gammaproteobacteria | Bacteria | restriction endonuclease S subunit | |
| 254780843 | Azotobacter vinelandii | Gammaproteobacteria | Bacteria | glucose/galactose transporter | |
| 254780845 | Thermobifida fusca | Actinobacteria (class) | Bacteria | ribonucleotide-diphosphate reductase subunit | related to mobile element in the |

| | | | | | |
|---|---|---|---|---|---|
| | | | | beta | genome ? |
| 254780918 | Methylobacter tundripaludum | Gammaproteobacteria | Bacteria | glycosyl transferase family protein | |
| 255764508 | Bordetella bronchiseptica | Betaproteobacteria | Bacteria | unknown | |
| 254780964 | Thermobifida fusca | Actinobacteria (class) | Bacteria | ribonucleotide-diphosphate reductase subunit beta | related to mobile element in the genome? |
| 254780982 | Photobacterium profundum | Gammaproteobacteria | Bacteria | homoserine lactone efflux protein | |
| 254780987 | Pseudomonas fluorescens | Gammaproteobacteria | Bacteria | transcriptional regulator | |
| 254781000 | Sodalis glossinidius | Gammaproteobacteria | Bacteria | tRNA (uracil-5-)-methyltransferase | |
| 254781016 | Neptuniibacter caesariensis | Gammaproteobacteria | Bacteria | diphosphomevalonate decarboxylase | |
| 254781017 | Thiomicrospira crunogena | Gammaproteobacteria | Bacteria | GHMP kinase | |
| 254781018 | Dichelobacter nodosus | Gammaproteobacteria | Bacteria | GHMP kinase | |
| 254781019 | Neptuniibacter caesariensis | Gammaproteobacteria | Bacteria | hydroxymethylglutaryl-coenzyme A synthase | |
| 254781020 | Neptuniibacter caesariensis | Gammaproteobacteria | Bacteria | isopentenyl pyrophosphate isomerase | |
| 255764509 | Neptuniibacter caesariensis | Gammaproteobacteria | Bacteria | HMG-CoA reductase | |
| 254781022 | Sodalis glossinidius | Gammaproteobacteria | Bacteria | membrane protein | |
| 254781025 | Rhodothermus marinus | Sphingobacteria | Bacteria | deoxyuridine triphosphate nucleotidohydrolase | |
| 254781068 | Legionella pneumophila | Gammaproteobacteria | Bacteria | glycoside hydrolase family protein | fragment |
| 254781070 | Candidatus Regiella insecticola | Gammaproteobacteria | Bacteria | glycosyl hydrolase | fragment |
| 254781071 | Candidatus Hamiltonella defensa | Gammaproteobacteria | Bacteria | glycosyl hydrolase | fragment |
| 254781114 | Acidovorax delafieldii | Betaproteobacteria | Bacteria | glycosyl transferase | |
| 254781147 | Xylella fastidiosa | Gammaproteobacteria | Bacteria | transcriptional regulator | |
| 254781149 | Selenomonas noxia | Negativicutes | Bacteria | replicative DNA helicase | |
| 254781169 | Vibrio vulnificus | Gammaproteobacteria | Bacteria | sugar transporter | |
| 254781170 | Serratia proteamaculans | Gammaproteobacteria | Bacteria | deoxyribodipyrimidine photolyase | |
| 254781171 | Legionella longbeachae | Gammaproteobacteria | Bacteria | Na+/H+-dicarboxylate symporter | |
| Proteins with close eukaryotic homologs (but they are not likely to really originated in eukaryota) | | | | | |
| **NCBI gi** | **Organism of the closest homolog** | **Class** | **Kingdom** | **Function prediction** | **Comments** |
| 254780480 | Ricinus communis | | Eukaryota | deoxycytidine triphosphate deaminase | only first abnormal |
| 254780640 | Ricinus communis | | Eukaryota | Chromosome segregation ATPase | fragment, too short to have sensible blast top hits |
| 254780519 | Gossypium hirsutum | | Eukaryota | flagellar biosynthesis protein FliQ | only the top hit is abnormal |
| Potential protein involve in the virulence effect of the pathogen | | | | | |
| **NCBI gi** | **Organism of the closest homolog** | **Class** | **Kingdom** | **Function prediction** | **Comments** |
| 254780898 | Thermodesulfovibrio yellowstonii | Nitrospira (class) | Bacteria | Protein serine/tyrosine phosphatase | has signal peptide |
| 254780995 | Photobacterium profundum | Gammaproteobacteria | Bacteria | Endonuclease/exonuclease/phosphatase family protein | has several similar proteins within the genome |
| 254781003 | Haliangium ochraceum | Deltaproteobacteria | Bacteria | Endonuclease/exonuclease/phosphatase family protein | has signal peptide, has several similar proteins within the genome |
| 254780607 | Yersinia frederiksenii | Gammaproteobacteria | Bacteria | unknown | has signal peptide |
| 254780965 | Stenotrophomonas maltophilia | Gammaproteobacteria | Bacteria | unknown | has signal peptide |
| 254780953 | Idiomarina baltica | Gammaproteobacteria | Bacteria | outer membrane lipoprotein | transmembrane protein |
| 254780291 | Photorhabdus luminescens | Gammaproteobacteria | Bacteria | unknown | transmembrane protein |
| 254780298 | Burkholderia sp. CCGE1001 | Betaproteobacteria | Bacteria | unknown | transmembrane protein |
| 254780338 | Arsenophonus nasoniae | Gammaproteobacteria | Bacteria | unknown | transmembrane protein |
| 254780449 | Buchnera aphidicola | Gammaproteobacteria | Bacteria | unknown | transmembrane protein |
